# Mechanisms of Pyrethroid Resistance in the Dengue Mosquito Vector, *Aedes aegypti*: Target Site Insensitivity, Penetration, and Metabolism

**DOI:** 10.1371/journal.pntd.0002948

**Published:** 2014-06-19

**Authors:** Shinji Kasai, Osamu Komagata, Kentaro Itokawa, Toshio Shono, Lee Ching Ng, Mutsuo Kobayashi, Takashi Tomita

**Affiliations:** 1 Department of Medical Entomology, National Institute of Infectious Diseases, Tokyo, Japan; 2 Environmental Health Institute, National Environmental Agency, Singapore; Mahidol University, Thailand

## Abstract

*Aedes aegypti* is the major vector of yellow and dengue fevers. After 10 generations of adult selection, an *A. aegypti* strain (SP) developed 1650-fold resistance to permethrin, which is one of the most widely used pyrethroid insecticides for mosquito control. SP larvae also developed 8790-fold resistance following selection of the adults. Prior to the selections, the frequencies of V1016G and F1534C mutations in domains II and III, respectively, of voltage-sensitive sodium channel (Vssc, the target site of pyrethroid insecticide) were 0.44 and 0.56, respectively. In contrast, only G1016 alleles were present after two permethrin selections, indicating that G1016 can more contribute to the insensitivity of Vssc than C1534. *In vivo* metabolism studies showed that the SP strain excreted permethrin metabolites more rapidly than a susceptible SMK strain. Pretreatment with piperonyl butoxide caused strong inhibition of excretion of permethrin metabolites, suggesting that cytochrome P450 monooxygenases (P450s) play an important role in resistance development. *In vitro* metabolism studies also indicated an association of P450s with resistance. Microarray analysis showed that multiple P450 genes were over expressed during the larval and adult stages in the SP strain. Following quantitative real time PCR, we focused on two P450 isoforms, CYP9M6 and CYP6BB2. Transcription levels of these P450s were well correlated with the rate of permethrin excretion and they were certainly capable of detoxifying permethrin to 4′-HO-permethrin. Over expression of *CYP9M6* was partially due to gene amplification. There was no significant difference in the rate of permethrin reduction from cuticle between SP and SMK strains.

## Author Summary

*Aedes aegypti* inhabits tropical and subtropical regions worldwide and is the major vector of dengue and yellow fevers, and a secondary vector of chikungunya fever. Dengue fever is epidemic in more than 110 countries and causes up to 100 million infections annually. As there is no efficient vaccine or medicine currently available, vector control remains the primary solution for reducing the number of cases of this disease and relies heavily on the use of insecticides. Intensive and long-term use of insecticides has resulted in the worldwide emergence of mosquitoes with resistance to these chemicals. Here we newly identified two P450s, which have the ability to detoxify pyrethroid insecticide and are over produced in the resistant *A. aegypti* strain. Our study showed there were at least four P450 isozymes associated with resistance and target site insensitivity. These findings may lead to the development of more accurate monitoring systems in the field and also assist to identify new target sites for insecticides that are effective against resistant insects.

## Introduction

The yellow fever mosquito *Aedes aegypti* inhabits tropical and subtropical regions worldwide and is the major vector of dengue fever (DF) and yellow fever. DF is a rapidly growing health issue, with the average annual number of cases being approximately 100 million and a 30-fold increase in the past 50 years [1], [2]. The disease is endemic in at least 112 countries, especially in south and Southeast Asia, and an estimated 2.5 billion people are currently living in risk areas [1]. At present, DF causes more illness and death than any other arbovirus disease in humans [3]-[5].

Successful population control of vector insects is the key to prevent transmission and epidemics of infectious diseases. This strategy relies heavily on insecticides, particularly pyrethroid, a popular class of insecticides with high and rapid toxic activity toward insects and low toxicity to mammals [6]. Pyrethroids are used to control and/or prevent adult mosquitoes by ultra-low volume sprays, thermal fogging, pyrethroid-impregnated nets, etc. However, many dengue endemic areas are now facing the problem of pyrethroid resistance due to frequent and intensive use of these chemicals [7]. Resistance of *A. aegypti* to pyrethroids has been reported from various countries [8]. Understanding the level and mechanism of resistance to insecticides is essential for developing appropriate vector control measures.

*A. aegypti* is present in most residential areas of Singapore. Despite a well-established national vector control program that includes community engagement, law enforcement, and inter-sectional coordination, Singapore continues to face the risk of DF resurgence [9]–[11]. During an outbreak in 2005, more than 14,000 DF cases were reported [12]. Since 2005, surveillance for dengue control has been based on four pillars: (1) case surveillance through mandatory notification of dengue cases to the Ministry of Health, by all medical practitioners; (2) vector surveillance through premises checks by vector control officers from National Environment Agency (NEA); (3) virus genotype surveillance at Environmental Health Institute of NEA; and (4) monitoring of other environmental parameters such as weather factors and population density. Clustering of cases and development of risk maps using the surveillance data allows prioritization of vector control operations. Though the program is largely based on source reduction and large scale fogging has not been conducted by the authorities since 2005, insecticides continue to be used by the authority for indoor misting in areas with dengue transmission, by private arrangement and household use of aerosol cans. Regular monitoring for insecticide has revealed that Singapore's *A. aegypti* larvae have developed resistance to synthetic pyrethroids, with resistance ratio to permethrin in the range of 29–47 times [13].

Generally, insecticide resistance in insects is caused by three major mechanisms: (1) reduced sensitivity of the target site, (2) reduced penetration of the insecticide due to altered cuticles, and (3) increased activity or level of detoxification enzyme(s). Insects develop resistance to insecticides by obtaining one or more of these mechanisms.

Amino acid substitution in the voltage-sensitive sodium channel (Vssc), the target site of DDT and pyrethroids, is the most well-understood mechanism conferring resistance against pyrethroid insecticides [14], [15]. This mechanism is called “knockdown resistance,” with its inherited trait, “*kdr*,” being first identified in the housefly as a leucine–phenylalanine substitution at position 1014 (L1014F) of Vssc. Recently, many other mutations that cause reduced sensitivity to Vssc have been identified from many arthropod species and are called either “*kdr*-like factor” or “knockdown resistance.” Knockdown resistance is unaffected by synergists that inhibit the activity of metabolic enzymes such as carboxyl esterases or cytochrome P450 monooxygenases (P450s). Several amino acid substitutions have been detected in *A. aegypti* Vssc: S989P, I1011M, I1011V, V1016G, V1016I, F1534C, and D1763Y [16]–[22]. Of these, only substitutions V1016G, V1016I, and F1534C have been shown to strongly correlate with pyrethroid resistance [17], [23]–[27]. The dominance of these three mutations on pyrethroid sensitivity, however, is not well understood.

Metabolism-mediated insecticide resistance is now considered a key mechanism in insects [7], [28], [29]. Three families of metabolic enzymes have been implicated in the metabolism of insecticides: esterases, glutathione transferases (GSTs), and P450s. The genome project of *A. aegypti* identified 26 esterases, 49 GSTs, and 160 P450s [30]. Identification of the specific enzymes involved in insecticide resistance, however, has proven challenging. P450s has been shown to be the metabolic enzyme most strongly linked to the development of pyrethroid resistance [31]–[33]. However, due to the large number of P450 genes and the structural similarity among different isoforms, identification of the isoform(s) associated with resistance has been difficult, with only few exceptions [34], [35]. Recently, microarray analysis that compares gene expression between susceptible- and resistant-strains unearthed candidate genes that confer insecticide resistance in different insect species [36]–[38]. Although molecular diagnosis of insecticide resistance that targets *Vssc* mutations is widely reported, such a system has not been reported for P450 genes in *Aedes* mosquitoes. Since insecticide resistance in a mosquito population could be concurrently affected by more than one mechanism, an accurate molecular diagnosis thus needs to consider the various possible mechanisms. Metabolism-mediated pyrethroid resistance of *A. aegypti* requires further study especially in the populations collected from Southeast Asia, the largest endemic area of DF.

Reduced cuticle penetration is the least understood mechanism among the three. Though it may have a primary role in resistance [39]–[41], it more often acts in combination with the other mechanism(s).

This study examined the mechanisms conferring pyrethroid resistance in an *A. a*egypti strain collected from Singapore and investigated three major mechanisms of resistance. The results are expected to lead to the development of more accurate resistance monitoring systems and contribute to the discovery of new insecticide target sites to overcome the challenge of insecticide resistant mosquitoes.

## Methods

### Ethics statement

Mice were used as the blood source for mosquitoes. This study complies with the guidelines for animal experiments performed at National Institute of Infectious Diseases, Japan. The protocol for the utility of mice was approved by the Animal Use Committee of National Institute of Infectious Diseases (registration numbers: 209052, 210044, 211031 and 112029).

### *A. aegypti* population and strains

A laboratory-susceptible reference strain of *A. aegypti*, SMK, was obtained from Sumitomo Chemical Co., Ltd. in 2009. This strain was originally from USA and has been maintained in the laboratory for at least 20 years without exposure to insecticides. The pyrethroid-resistant population, SPS_0_, was collected from Singapore in 2009. The SPS_0_ population was selected by exposure to permethrin for 10 generations as described below and the SP strain ( = SPS_10_) was established. Permethrin doses used for the selections are listed in Table 1. Larvae were fed a ground diet of insect food (Oriental Yeast Co., Ltd., Tokyo, Japan), and the adults were maintained on 10% sucrose. Females were given blood meals from mice. Both larvae and adults were reared at 27 ± 1°C with a photoperiod of 16∶8 (L∶D) h.

**Table 1:** Selection of the SP strain of *Aedes aegypti* in the laboratory by permethrin.

Selection No.	Selected population	Gender	Permethrin dose (ng/mosquito)	No. mosquitoes treated	Mortality (%)
1	SPS_0_G3	♂	10	600	52.0
		♀	60	600	62.8
2	SPS_1_G1	♂	27	480	60.6
		♀	177	480	76.9
3	SPS_2_G1	♂	44	480	57.1
		♀	333	490	85.3
4	SPS_3_G1	♂	61	513	31.8
		♀	444	551	69.1
5	SPS_4_G1	♂	111	515	74.0
		♀	444	524	50.2
6	SPS_5_G2	♂	167	629	89.2
		♀	821	482	79.0
7	SPS_6_G1	♂	167	527	71.3
		♀	821	510	78.4
8	SPS_7_G1	♂	167	583	82.7
		♀	821	613	86.8
9	SPS_8_G1	♂	167	542	65.1
		♀	821	512	73.6
10	SPS_9_G1	♂	167	529	46.3
		♀	821	547	45.2

The subscript numbers in the population name indicate the permethrin selections in the population. For example, SPS_5_G2 indicates the 2nd generation of SPS5 which was selected by permethrin for five generations.

### Chemicals

Three technical grade insecticides were used: permethrin (94.1%, Sumitomo Chemical Co., Ltd., Osaka, Japan), temephos (94.9%, Wako Chemical Pure Industries, Ltd., Osaka, Japan), and pirimiphos methyl (98.0%, Wako Chemical Pure Industries, Ltd., Osaka, Japan). An inhibitor of P450s, piperonyl butoxide (PBO, 98.0%, Wako Chemical Pure Industries, Ltd., Osaka, Japan), was used for the adult and larval bioassays. [^14^C]-(1RS)-trans-permethrin (specific activity, 4.44 GBq/m mol, purity >99%) labeled at the phenoxy benzyl group and 4′-hydroxy-3-phenoxy-benzyl 3-(2,2-dichlorovinyl)-2,2-dimethylcyclopropane-carboxylate (4′-HO-permethrin) were generously provided by Sumitomo Chemical Co., Ltd. (Osaka, Japan). [^14^C]-(1RS)-trans-permethrin was purified by thin layer chromatography (TLC) according to the modified method described by Shono et al. [42]. We used a solvent system of toluene/carbon tetrachloride (1∶1) instead of benzene/carbon tetrachloride (1∶1) in order to separate trans-permethrin from other degraded compounds.

### Permethrin selections and adult bioassay

Three- to seven-day-old adult male and virgin females were used for the permethrin selections. In order to avoid measurement error caused by insect locomotive activity, we used a topical application method, instead of filter paper method, for pyrethroid selections and adult bioassays. The mosquitoes were anaesthetized with CO_2_ and placed in a 200-ml Erlenmeyer glass flask. They were then re-anaesthetized for 3 min with 100 µl diethyl ether absorbed onto cotton. A volume of 0.22 µl permethrin in acetone solution was dropped onto the thoracic notum of each mosquito with a Hamilton repeating dispenser PB-600 equipped with 10 µl syringe 701SNR (Hamilton, Reno, NV, USA). The insects were placed in groups of 40 in paper cups sealed with a nylon mesh. Water-absorbed cotton was placed on the top of the mesh. Approximately 1000 mosquitoes were selected from each generation (Table 1). The doses of permethrin used for the selections were determined by a preliminary experiment for each gender. Twenty-four hours after treatment, all the mosquitoes were put into a cage, provided with sucrose water, and maintained for the next generation. A bioassay to monitor permethrin susceptibility was conducted for each selected generation as described below.

Adult bioassays were conducted by topical application using 3- to 7-day-old female mosquitoes and consisted of at least five doses of permethrin causing >0% and <100% mortality. All bioassays were run at 25°C with 40 mosquitoes per dose. Mortality was assessed 24 h after treatment, with mosquitoes that could not stand on the bottom of cup counted as dead. The LD_50_ values for each strain were calculated using log-probit mortality regression analysis [43]. To estimate the contribution of P450s to resistance, a 0.22 µl (5 µg) aliquot of piperonyl butoxide (PBO) was placed on the thoracic notum of the mosquitoes 24 h prior to dosing with permethrin. A preliminary experiment confirmed that this PBO dose did not kill any of the mosquitoes. The resistance ratios were calculated based on the ratio of LD_50_ values for the SMK and SP strains.

### Crossing

To obtain F1 progeny, 100 virgin females and 100 males were randomly crossed and the offspring was used in the permethrin bioassays. BC1 progeny was also obtained by backcrossing 100 females F1 (SP♀ × SMK♂) and 100 SMK♂. Opposite backcrossing was also conducted. Permethrin bioassays were conducted for both BC1 progenies as described above.

### Larval bioassays

Larval bioassays were carried out as described elsewhere [44]. Twenty early fourth instar larvae were exposed to different concentrations of insecticides in 50 ml distilled water and mortality was counted after 24 h exposure at 25°C. Alcohol solutions of the insecticides (×200 of final conc., 250 µl) were added to the water. For estimating synergistic effects, the larvae were treated with a sublethal dose of PBO (5 µg/ml) in combination with permethrin. Mortality was assessed 24 h after treatment, with larvae that could not swim to the surface counted as dead. Three replications were run for each insecticide concentration, and the LC_50_ values for each strain and insecticide were calculated using log-probit mortality regression analysis [43].

### Genomic DNA extraction

DNA was extracted from individual male mosquitoes using the REDExtract-N-Amp Tissue PCR Kit (Sigma, MO, USA). We used male mosquitoes to avoid gene contamination derived from sperm DNA in females. In *A. aegypti*, sex is determined by M-locus which is mapped on the 1st chromosome [45]. Since *Vssc* gene is located on the 3rd chromosome [24], theoretically, genotyping of *Vssc* genes from males does not cause any gender bias. The extraction and tissue preparation solutions were mixed, and each mosquito was homogenized in a 200-µl PCR tube for 1 min at 1500 rpm using a shaking homogenizer (MM300, Retsch Co., Haan, Germany) in 62.5 µl of the mixture containing a zirconia ball (4 mm in diameter). After homogenization, the samples were incubated at room temperature for 30 min, followed by incubation at 95°C for 3 min. A 50-µl aliquot of neutralization solution was then added and mixed by vortexing. The extracted mixture was stored at −20°C.

### Genotyping of knockdown resistance gene

Male mosquitoes were genotyped for their *Vssc* alleles as described previously [46]. We targeted six amino acid positions to identify the candidate(s) for knockdown resistance: the typical kdr, L1014F, and five amino acid positions in Vssc previously identified from pyrethroid resistant *A. aegypti* (i.e., S989P, I1011M or V, V1016G or I, F1534C, and D1763Y) [16], [17], [19], [20], [22]. In this study we numbered the amino acid position according to the sequence of the most abundant splice variant of the house fly *Vssc* (GenBank accession nos. AAB47604 and AAB47605). Partial DNA fragments of domains II, III, and IV were amplified by PCR using TaKaRa Ex Taq Hot Start Version (Takara Bio, Shiga, Japan) and three primer sets: AaSCF20 and AaSCR21 (for domain II), AaSCF7 and AaSCR7 (for domain III), and AlSCF6 and AlSCR8 (for domain IV). The primer sequences are listed in Table S1. The cycling conditions for PCR were as follows: initial denaturation at 95°C for 5 min, followed by 35 cycles of 94°C for 30 s, 55°C for 30 s, 72°C for 1 min, and a final extension step at 72°C for 5 min. The PCR products were then treated with illustra ExoStar (GE Healthcare UK Ltd., Little Chalfont, England) to remove unincorporated primers and dNTPs, followed by sequencing with the following primers: AaSCF3 (forward primer for domain II), AaSCR22 (reverse primer for domain II), AaSCR8 (reverse primer for domain III), and AlSCF7 (forward primer for domain IV) using an ABI 3130 Genetic Analyzer (Applied Biosystems, Foster City, CA, USA). The sequences were assembled and aligned using the GENETYX software (GENETYX Corporation, Tokyo, Japan).

### *In vivo* metabolism

A dose of 600 dpm (ca 0.88 ng) [^14^C]-permethrin in 0.22 µl acetone was administered to the thoracic notum of female mosquitoes. Each treated mosquito was isolated in a scintillation vial (6 ml) with a water-absorbed small cotton pad. The mosquitoes were anaesthetized with diethyl ether at 0.75, 1.5, 3, 6, 12, 24, and 48 h after treatment and rinsed in methanol (0.5 ml × 2). The methanol was then added to 4 ml of scintillation cocktail Ultima Gold fluid (PerkinElmer Inc., Waltham, MA, USA) and analyzed in a liquid scintillation counter LSC-3100II (Hitachi Aloka Medical, Ltd., Tokyo, Japan). To study effect of synergist on permethrin penetration, PBO (5 µg/♀) was placed on the thoracic notum or thoracic sternum 1 h before permethrin treatment. To assess the effects of solvent on permethrin penetration, 0.22 µl of acetone was applied to the thoracic notum 1 h prior to permethrin treatment (negative control). Each time point was replicated four times, with 32 mosquitoes (four mosquitoes × eight time points) being used for each experiment.

After being rinsed in methanol, each mosquito was placed into a 2-ml safe-lock tube (Eppendorf Co., Ltd., Hamburg, Germany) with a zirconia ball (4 mm in diameter) and homogenized for 1 min at 1500 rpm with 0.5 ml of scintillation cocktail Ultima Gold fluid (PerkinElmer Inc., Waltham, MA, USA) in a shaking homogenizer (MM300, Retsch Co., Haan, Germany). The homogenized solution was transferred to a scintillation vial and rinsed again with 1 ml of scintillation cocktail. An additional 2.5-ml of cocktail was added to the vial (total volume 4 ml) and the radioactivity inside the mosquito was counted using a liquid scintillation counter.

To measure the rate of permethrin excretion, the holding vial was extracted with 4 ml of scintillation cocktail and the radioactivity was counted using a liquid scintillation counter. Each time point was replicated four times (four individual mosquitoes) in each experiment. The rate constants for the *in vivo* dynamics of topically applied permethrin were calculated using the linear one compartment model described previously [47], [48].

To identify the metabolites excreted into the vial, 10 SP females had ca 17 ng (11,800 dpm) of [^14^C]-trans-permethrin in 0.44 µl acetone applied to the thoracic notum and were then placed into 20 ml glass vials. Three replicates were conducted. Forty-eight hours after treatment, the mosquitoes were anaesthetized with diethyl ether, removed from the vial, and the residual isotope in the vial was extracted with methanol (3 ml × 2). The methanol was evaporated by N_2_ gas, and aliquots (5000 dpm) were spotted onto silica gel plates 60 F254 (HPLC, 0.2 mm, Merck KGaA, Damstadt, Germany), followed by development in a solvent of toluene/ethyl acetate (6∶1). The HPTLC plate was auto-radiographed, scanned with a Bio-Imaging Analyzer BAS2500 (Fuji Photo Film, Tokyo, Japan), and the signal intensities of each metabolite were quantified. Two-dimensional development was conducted for water soluble metabolites using a solvent of chloroform/methanol/water (65∶25∶4) and auto-radiographed as described above. Unlabeled authentic 4′-HO-permethrin was co-chromatographed and identified by viewing under ultraviolet light at 254 nm.

### Preparation of microsomes

Microsomes were prepared from the abdomens of 3-7-old female mosquitoes using a modified procedure as described previously [49]. Mosquitoes were anaesthetized with CO_2_ and transferred to a glass Petri dish placed on crushed ice. Two hundred abdomens were separated from the thoraces of mosquitoes using two sets of forceps and put into a glass container on ice containing 5 ml of homogenization buffer [49]. The collected abdomens were homogenized with glass–teflon Dounce homogenizer for 20 strokes using homogenizing mixer HK-1 (Asone, Osaka, Japan) and filtered through a layer of nylon wool. Homogenates were then centrifuged at 4°C at 10,000 xg for 15 min in an Eppendorf 5804R (Eppendorf Co., Ltd., Hamburg, Germany) equipped with a F-34-6-38 rotor. The 10,000 xg supernatant was centrifuged again at 4°C at 100,000 xg for 1 h in a Beckman Optima MAX-XP (Beckman Coulter Inc., Brea, CA, USA) equipped with a MLS-50 rotor. The microsomal pellets were resuspended by homogenizing in 2 ml resuspension buffer [49]. The supernatant of the 100,000 xg centrifugation was also collected and used for an *in vitro* metabolism study.

### *In vitro* metabolism

*In vitro* metabolism was studied as described previously with some modifications [44]. The 2-ml reaction mixture contained incubation buffer [0.1 M sodium phosphate buffer (pH 7.5) containing 1 mM EDTA, 0.1 mM DTT, and 1 mM PMSF dissolved in ethylene glycol monomethyl ether], microsomes or supernatant equivalent to 10 abdomens, 0.2 ml of 10 mM β-NADPH, and 100,000 dpm (0.147 µg) of [^14^C]-trans-permethrin in 10 µl ethanol. An incubation mixture without β-NADPH served as the control. For the inhibition study, 10 µl of PBO (20 mM) or 4′-HO-permethrin (0.5, 5.0, and 10.0 µg/ml equivalent to ×7, ×70, and ×140 final concentration of permethrin, respectively) in ethanol was added to the mixture. The mixtures were incubated for 5, 30, 60, 120, and 360 min at 25°C with shaking, and then incubation was terminated by adding 0.2 ml of 1 N HCl, followed by 1 g of (NH_4_)_2_SO_4_. Each sample was extracted with diethyl ether (4 ml × 3), by vortexing for 1 min and then centrifuged at 4000 xg for 1 min, evaporated by N_2_ gas, and redissolved in 100 µl methanol. Aliquots of the extracted compounds (5,000 dpm) were spotted on HPTLC plates and developed in a solvent of toluene/ethyl acetate (6∶1). The HPTLC plate was auto-radiographed, scanned with a Bio-Imaging Analyzer, and the signal intensities were measured as described above. Each time point for each strain was replicated three times using different enzyme sources. All the experiments involved the simultaneous use of the SP and SMK strains. The metabolites being stuck at the spotting position of the plate after development were collected, extracted with methanol (4 ml × 2), evaporated, and then spotted onto HPTLC plates. The high polar compounds were developed with a solvent of chloroform/methanol/water (65∶25∶4) and auto-radiographed as described above.

### Microarrays

The microarray used in this study was designed using the Agilent eArray platform (Agilent Technologies, CA, USA) and contained 60 mer oligo-probes representing >15,000 *A. aegypti* Liverpool transcripts identified in the genome project (https://www.vectorbase.org/content/aedes-aegypti-liverpooltranscriptsaaegl13fagz). Each probe was spotted at least two times at different positions on each array. A probe of each P450 gene was spotted six times. Microarrays in a 4 × 44 k format were constructed using contract manufacturing carried out by Agilent Technologies. The entire design of the 44 k array used in this study is available from the NCBI Gene Expression Omnibus (GEO) site as accession # GPL17604.

The strains were reared in parallel in order to minimize variation resulting from breeding conditions. For each life stage (larvae, adult males and females), four RNA sources were prepared from each of the SP and SMK strains reared in different trays and cages ( =  four biological replicates). Total RNA was extracted from 10 fourth instar larvae or 10 three-day-old adults using ISOGEN (Nippon Gene Co., Ltd., Tokyo, Japan). Genomic DNA was removed by digesting the total RNA samples with DNase I using TURBO DNase (Life Technologies Co., Carlsbad, CA, USA). The quality and quantity of total RNA were assessed by spectrophotometry using Nanodrop ND-1000 (Thermo Fisher Scientific Inc., Waltham, MA, USA) and a bioanalyzer MultiNA (Shimadzu Co., Kyoto, Japan). The purified RNA was mixed with the internal control RNA supplied in a RNA Spike-In Kit (One-color, Agilent Technologies). Fluorescein-labeled cRNA were synthesized via a double-stranded cDNA intermediate using a Low Input Quick Amp Labeling Kit (Agilent Technologies). The cRNA derived from the SP and SMK strains were differentially labeled with cyanine-3 (Cy-3) dye-conjugating CTP (PerkinElmer Inc., Waltham, MA, USA). cRNA was then purified with Qiagen RNeasy Mini Kit (Qiagen, Venlo, Netherlands), and the overall efficacy of cRNA synthesis and fluorescein-labeling was measured using a Nanodrop ND-1000 spectrophotometer. The labeled cRNA were pooled and hybridized to microarray probes in a hybridization oven at 65°C for 17 h under rotation at 10 rpm using a Gene Expression Hybridization Kit (Agilent Technologies, Santa Clara, CA, USA). After hybridization washing, the fluorescent dyes were stabilized against ozone oxidization with the Gene Expression Wash Buffer (Agilent Technologies) and the microarray plate was then dried in nitrogen gas. The fluorescence of Cy-3 on each spot was scanned using a DNA Microarray Scanner G565BA (Agilent Technologies, Santa Clara, CA, USA). Spot identification and quantification were performed using Feature Extraction Software v. 7.5 (Agilent Technologies, Santa Clara, CA, USA) in the default setting. A linear and LOWESS algorithm (a combination of the linear method and traditional LOWESS method) was used for dye normalization. Flagged spots (such as saturated, low intensity, and statistical outlier) were ignored in the final data analysis.

The ratio of transcription levels for each gene in the microarray experiment for the SP and SMK strains was called the “microarray ratio.” A representative microarray ratio in each hybridization experiment was expressed as the average of all spots for a gene on an array, where the spot number for a gene is calculated as “unique probe number” x “replicating spot number (ideally n  =  4).” The *P*-value for every spot was calculated by Agilent's Universal Error Model. The raw results of the 10 microarray hybridizations are available from GEO (series accession# GSE50069). The genes over expressed in the SP strain were selected using a cut-off of >3-fold relative change in expression and a *P*<0.01.

### Sequencing analysis of P450 genes

In order to design primers for real time quantitative PCR, a partial or full length sequence of the P450 genes (*CYP6BB2*, *CYP6Z7*, *CYP6Z8*, *CYP9M4*, *CYP9M5*, *CYP9M6*, *CYP9M7*) and an internal control gene (Ribosomal protein S3 gene, *RPS3*) were amplified and sequenced from eight male mosquitoes for each of the SP and SMK strains. We focused on these P450 genes according to the following criteria: over expressed in females (>5) and in males (>3) in SP relative to SMK in microarray analysis. *CYP9M7* was also analyzed as it forms a gene cluster with *CYP9M4*, *CYP9M5*, and *CYP9M6* within 65 kbp on the same supercontig (1.29) and is structurally related to these genes. The primer sequences and their regions are shown in Table S1 and Figure S1. Genomic DNA was extracted from individual male mosquitoes as described above. The PCR was conducted using high fidelity polymerase (PrimeSTAR GXL DNA Polymerase; Takara Bio Inc., Shiga, Japan), treated with illustra ExoStar (GE Healthcare UK Ltd., Little Chalfont, England) to remove unincorporated primers and dNTPs, followed by direct sequencing with the primers listed in Table S1. Because two haplotypes of *CYP9M6*, namely *CYP9M6v1* and *CYP9M6v2*, were identified from the SP strain, the primer sets, 9M6F31/9M6R21 and 9M6F23/9M6R35 for CYP9M6v1, and 9M6F30/9M6R22 and 9M6F18/9M6R35 for CYP9M6v2 were used to amplify each variant, followed by direct sequencing of the PCR products (Figure S2 and Table S1).

### Quantitative real-time PCR

cDNA was synthesized using total RNA isolated for microarray analysis, and the QT' primer (Table S1) and reverse transcriptase ReverTra Ace (Toyobo, Osaka, Japan) according to the manufacturer's instructions. Real time quantitative PCR was performed using a PikoReal Real Time PCR System (Thermo Fisher Scientific Inc., Waltham, MA, USA). The PCR primers were designed using Primer Express software (Applied Biosystems, Foster City, CA, USA). Each PCR reaction of 10 µl final volume contained 5 µl SYBR Premix Ex Taq II (Takara Bio Inc., Shiga, Japan), 1 µl cDNA (equivalent to 10 ng total RNA), 0.4 µl of each forward and reverse primer (10 µM, Table S1), and 3.2 µl ddH2O. The PCR reactions were performed under the following conditions: 95°C for 2 min, followed by 40 cycles of 95°C for 10 s, and 60°C for 30 s. The 2^−ΔΔCt^ method [50] was used to quantify the relative expression level of P450s, with *RPS3* gene acting as the internal control. For each gene analyzed, serial dilutions of cDNA showed that the efficacy of amplification for all P450 genes and the *RPS3* gene were >0.998. Three replicates of the PCR reactions were performed for each sample (technical replications) and each experiment was repeated four times using an independent RNA source (biological replications).

### Identification of gene copy number

Gene copy number was determined by quantitative PCR using the same primer sets described above. Genomic DNA was individually extracted from eight virgin females of each strain using Get Pure DNA Kit-Cell, Tissue (Dojindo Molecular Technologies, Inc., Kumamoto, Japan) according to the manufacturer's instructions. The quantity and quality of the DNA was assessed using a spectrophotometer Nanodrop ND-1000 (Thermo Fisher Scientific Inc., MA, USA). The DNA samples were diluted to 100 ng/µl and 1 µl used as a template. Data were normalized using *RPS3* gene and the exact single gene copy number reported by the genome project. Three replicates of the PCR reactions were performed for each sample (technical replications) and each experiment was conducted using 8 individual DNA source (biological replications).

### Association between permethrin excretion and *CYP9M6* and *CYP6BB2* genotypes

One hundred virgin males and females of the SP and SMK strains were cross-mated. The offspring were then interbred to obtain F2 mosquitoes. Three-day-old F2 females were dosed with a sub-lethal amount of [^14^C]-permethrin (ca 600 dpm, 0.88 ng) as described above. Each treated mosquito was isolated in a scintillation vial and the amount of radioisotope excreted was quantified 24 h later. Genomic DNA was isolated from six legs of each mosquito as described above using a REDExtract-N-Amp Tissue PCR Kit. Both *CYP9M6* and *CYP6BB2* were genotyped. Genotyping of *CYP9M6* was carried by real time quantitative PCR using three sets of primer: 9M6F88/9M6R89 (common), 9M6F95/9M6R97 (specific to SP), and 9M6F96/9M6R97 (specific to SMK). The primer sequences are listed in Table S1. Genotyping of *CYP6BB2* was carried out by analysis of the melting curve of different peaks in the post-PCR assay. PCR was performed using a TaKaRa Ex Taq Hot Start Version (Takara Bio, Shiga, Japan), 6BB2F21/6BB2R22 primers, 0.5 µl EvaGreen (Biotium, Inc., Hayward, CA, USA), and 1 µl genomic DNA in 10 µl of reaction mixture. The cycling conditions for PCR were as follows: initial denaturation of 95°C for 1 min, followed by 50 cycles of 95°C for 10 s and 60°C for 5 s. This reaction produced 43 bp PCR products (Figure S3). The PCR and dissociation analysis were performed using a PikoReal Real Time PCR System (Thermo Fisher Scientific Inc., Waltham, MA, USA). Total RNA was isolated from the remaining bodies of the mosquitoes and cDNA was synthesized as described above. Real time quantitative PCR was performed for *CYP9M6* and *CYP6BB2* to quantify the levels of transcription of these genes, as described above. Forty-two females (24 females for each crossing) were used for the analysis.

### Expression of P450s, reductase, and b_5_ in Sf9 cells

Full-length cDNAs encoding *CYP9M6* and *CYP6BB2* from the SP strain were cloned into the pPSC8 protein expression vector (Wako Chemical Pure Industries, Ltd., Osaka, Japan) using unique restriction sites positioned in the vector regions (XbaI and PstI for *CYP9M6* and XbaI and KpnI for *CYP6BB2*). Because we identified two variants for *CYP9M6* in the SP strain (*CYP9M6v1* and *CYP9m6v2*), these two genes were amplified using primers 9M6F85/9M6R86 for *CYP9M6v1* and 9M6F85/9M6R87 for *CYP9M6v2*. For amplification of *CYP6BB2* cDNA, 6BB2F19 and 6BB2R20 primers were used and cloned into the pPSC8 vector (Table S1 and Figure S1). A 200-µl aliquot of Sf900II medium (Life Technologies Co., CA, USA) containing 2 µg of the vectors (pPSC8/CYP9M6 or pPSC8/CYP6BB2), 85 ng Linear AcNPV (the baculovirus Autographa californica nuclear Polyhedrosis Virus), 5 µl Insect GeneJuice Transfection Reagent (Merck KGaA, Damstadt, Germany) was transfected to expressSF+ cells (1 × 10^6^ cells in 25 cm^2^ flask, Wako Chemical Pure Industries, Ltd., Osaka, Japan). The infected cells were incubated at 28°C for five days, centrifuged at 3000 xg for 30 min at 4°C, and the supernatant used for protein expression.

Full-length cDNA encoding NADPH cytochrome P450 reductase (PRE) and cytochrome b_5_ cDNAs was also amplified with the aegREDF9/aegREDR5 and aegb5F1/aegb5R2 primer sets, respectively, and then cloned into the pFastBac1 vector (Life Technologies Co., Carlsbad, CA, USA) using multiple cloning sites (StuI and XbaI for PRE and EcoRI and XbaI for b_5_). Recombinant constructs (pFastBac1/PRE and pFastBac1/b5) were used to transform MAX Efficiency DH10Bac competent cells (Life Technologies Co., Carlsbad, CA, USA). Recombinant bacmid DNA was isolated according to the manufacturer's instructions. The Sf9 cells were infected with recombinant baculovirus in Grace's Insect Medium with Cellfectin II (Life Technologies Co., Carlsbad, CA, USA), according to the manufacturer's instructions.

For preparation of cell lysates expressing P450, 50 ml of confluent cells were infected in a 125-ml flask, and incubated at 28°C with shaking at 130 rpm. Twenty-four hours after infection, hemin (in 50% ethanol and 0.1 N NaOH) was added to the media to a final concentration of 2 µg/ml, followed by further incubation for 48 hours. The cells were then centrifuged at 3000 xg at 4°C for 30 min and the pellets were used for the *in vitro* permethrin metabolism study.

### Metabolism of permethrin by heterologously expressed CYP9M6 and CYP6BB2

The cell pellets prepared as described above were resuspended in 5 ml of homogenization buffer [49] and sonicated with an ultrasonic processor Sonics Vibra-Cell VCX130 (Sonics & Materials, Inc., Newtown, CT, USA) for 2 min on ice water (pulse on 10 s, pulse off 10 s, with 20% amplitude). The homogenates were centrifuged at 10,000 xg for 15 min and the supernatant was then centrifuged at 100,000 xg for 1 h as described above for the preparation of microsomes. The microsomal pellets were resuspended by homogenizing in 2 ml of resuspension buffer [49], and the protein concentration was determined using Bradford's reagent (Coomassie Plus Protein Assay; Thermo Fisher Scientific Inc., Waltham, MA, USA). The *in vitro* permethrin metabolism study was conducted as described above using 7 mg of microsomal proteins. The experiment was replicated for tree times.

## Results

### Establishment of a permethrin-resistant strain

**Figure 1:**
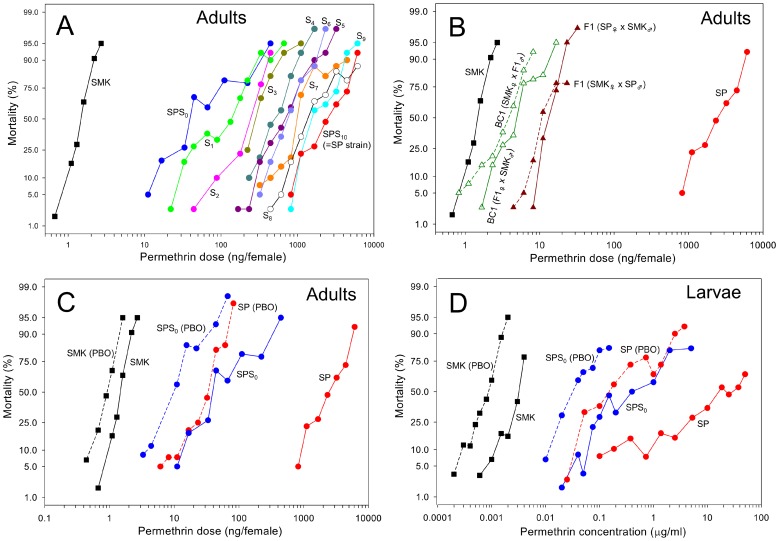
Log dose-probit mortality lines of *Aedes aegypti*. (A) The development of permethrin resistance in a field-collected population of adult *A. aegypti* under laboratory selection conditions. SPS_0_ is a field-collected population prior to permethrin selection. SPS_10_ is the strain established by permethrin selection for 10 generations and is designated as SP. SMK is a susceptible reference strain. (B) Dose–mortality lines of adult F1 (SP_♀_ × SMK_♂_ or SMK_♀_ × SP_♂_) and BC1 (F1_♀_ × SMK_♂_ or SMK_♀_ × F1_♂_) showing the inheritance of SP resistance. (C) Synergistic effects of PBO on permethrin toxicity in adults. (D) Susceptibility of larvae to permethrin and the synergistic effects of PBO.

A field population of *A. aegypti* collected from Singapore was selected for permethrin resistance by subjecting a wild type population (SPS_0_) to the chemical for 10 generations (Table 1). Initially, the resistance ratio (RR) of SPS_0_ was 35-fold. The RR rose rapidly over the generations and the established SP strain ( = SPS_10_) developed 1650-fold resistance after 10 generations of permethrin selection (Figure 1A). The RR for each generation is summarized in Table 2. To determine the degree of dominance of the resistance [51], the SP strain was crossed with a susceptible reference strain (SMK) and bioassays were performed on the progenies. The degrees of dominance for F1 (SP_♀_ × SMK_♂_) and F1 (SMK_♀_ × SP_♂_) were −0.337 and −0.383, respectively (Figure 1B).

**Table 2:** Toxicity of permethrin and pirimiphos methyl by topical application to adult *Aedes aegypti* and the synergistic effects of PBO.

Insecticide	Population or strain	n^a^	Slope ± SE	LD_50_ (ng/♀)	95% CL	SR^b^	RR^c^
Permethrin	SMK	354	7.0 ± 0.64	1.5	1.4–1.6		1.0
	SPS_0_	480	2.0 ± 0.14	52	37–74		35
	SPS_1_	440	2.2 ± 0.18	106	92–123		71
	SPS_2_	199	3.9 ± 0.41	212	157–278		141
	SPS_3_	200	4.7 ± 0.51	318	212–443		212
	SPS_4_	280	4.2 ± 0.37	509	461–562		339
	SPS_5_	400	3.2 ± 0.26	685	610–769		457
	SPS_6_	280	7.4 ± 0.75	757	684–838		505
	SPS_7_	360	5.2 ± 0.50	1090	959–1240		727
	SPS_8_	360	4.7 ± 0.46	1650	1440–1890		1100
	SPS_9_	280	6.4 ± 0.65	1900	1690–2130		1270
	SPS_10_ ( = SP)	280	5.0 ± 0.59	2470	2160–2840		1650
	F1(SP_♀_ × SMK_♂_)	200	11.8 ± 1.5	14	13–15		9.3
	F1(SMK_♀_ × SP_♂_)	240	7.8 ± 0.84	12	11–14		8.0
	BC1(SMK_♀_x F1(SP_♂_ × SMK_♀_)_♂_)	320	5.8 ± 0.62	3.6	3.2–4.0		2.4
	BC1(F1(SP_♀_ × SMK_♂_)_♀_ × SMK_♂_)	320	5.9 ± 0.62	4.9	4.4–5.5		3.3
Permethrin +PBO^d^	SMK	300	6.0 ± 0.55	0.89	0.83–0.95	1.7	1.0
	SPS_0_	420	2.8 ± 0.22	9.8	7.3–13	5.3	11
	SPS_10_ ( = SP)	360	5.8 ± 0.57	30	26–34	82	34
	SP_♀_ × SMK_♂_	200	9.7 ± 1.4	3.3	3.0–3.6	4.2	3.7
	SMK_♀ ×_ SP_♂_	240	10.5 ± 1.2	2.3	2.1–2.5	5.2	2.6
Pirimiphos methyl	SMK	360	10.5 ± 0.94	7.7	7.0–8.4		1.0
	SPS_0_	359	6.5 ± 0.56	20	19–21		2.6
	SPS_10_ ( = SP)	200	16.2 ± 2.2	38	35–41		4.9

a n, number of females tested.

b SR, synergistic ratio  =  LD_50_ (without PBO)/LD_50_ (with PBO).

c RR, resistance ratio  =  LD_50_ (each population)/LD_50_ (SMK).

d PBO (5 µg/mosquito) was administered 1 h prior to permethrin treatment.

The toxicity of permethrin was markedly increased in the SP strain by pretreatment with the P450 inhibitor piperonyl butoxide (PBO), with the RR decreasing from 1650- to 33-fold. The synergistic ratio (SR) of the SP and SMK strains was 1.7 and 83, respectively, indicating a significant role of P450s in permethrin resistance of SP (Figure 1C and Table 2). The low SR of SPS_0_, at only 5.3, suggested that the P450-mediated resistance mechanism was initiated or enhanced during the process of permethrin selection. We observed an approximately three-fold difference in the LD_50_s between SPS_0_ (9.8 ng/female) and SP (30 ng/female) in the presence of PBO. This suggested that a mechanism, other than P450s also contributed to the development of high level of permethrin resistance in SP (Table 2). The SP strain exhibited a 4.9-fold cross-resistance to pirimiphos methyl after permethrin selection.

Although the laboratory selections were conducted during the adult stage, larvae of the SP strain also developed a high level of resistance to permethrin (Figure 1D and Table 3), from RR of 160-fold for SPS_0_ to 8790-fold for SP. PBO also increased the toxicity of permethrin in the larval stage. The SRs of SMK, SPS_0_, and SP were 3.5, 8.7, and 126, respectively. The SP strain showed a low level of resistance to organophosphates, with the RRs of larvae to temephos and pirimiphos methyl being 1.5- and 4.2-fold, respectively (Table 3).

**Table 3:** Toxicity of permethrin, temephos, and pirimiphos methyl to larval *Aedes aegypti* and the synergistic effects of PBO.

Insecticide	Population or strain	n^a^	Slope±SE	LC_50_ (µg/ml)	95% CL	SR^b^	RR^c^
Permethrin	SMK	372	4.0 ± 0.32	0.0028	0.0021–0.0038		1.0
	SPS_0_	655	1.2 ± 0.08	0.45	0.31–0.72		160
	SPS_10_ ( = SP)	472	1.2 ± 0.15	24.6	15.6–45.3		8790
Permethrin + PBO^d^	SMK	539	3.5 ± 0.27	0.00079	0.00073–0.00087	3.5	1.0
	SPS_0_	421	2.2 ± 0.18	0.039	0.033–0.044	11.5	49
	SPS_10_ ( = SP)	398	1.9 ± 0.20	0.20	0.14–0.26	123	253
Temephos	SMK	313	10 ± 0.85	0.016	0.015–0.017		1.0
	SPS_0_	316	7.0 ± 0.71	0.019	0.016–0.023		1.2
	SPS_10_ ( = SP)	320	7.7 ± 0.94	0.024	0.022–0.027		1.5
Pirimiphos methyl	SMK	506	4.0 ± 0.39	0.031	0.026–0.043		1.0
	SPS_0_	312	5.2 ± 0.50	0.067	0.063–0.073		2.2
	SPS_10_ ( = SP)	300	10 ± 1.1	0.13	0.12–0.14		4.2

a n, number of larvae tested.

b SR, synergistic ratio  =  LD_50_ (without PBO)/LD_50_ (with PBO).

c RR, resistance ratio  =  LD_50_ (each population)/LD_50_ (SMK).

d PBO (5 µg/ml) was administered 1 h prior to permethrin treatment.

### Frequencies of knockdown resistance type alleles

We genotyped five positions of *Vssc*, which potentially cause decreased sensitivity to pyrethroid insecticides. Sequencing of the partial DNA of *Vssc* identified three amino acid substitutions in the SPS_0_ population compared with the SMK strains: S989P, V1016G, and F1534C. Of these substitutions, P989 and G1016 always appeared together, indicating that these polymorphisms are located on the same haplotype. All mosquitoes in the SPS_0_ population possessed either the P989+G1016 or C1534 haplotypes, with a frequency of 44% and 56%, respectively. After the first selection, the frequency of P989+G1016 increased to 79%, and following the second selection, the C1534 haplotypes were no longer detected (Table 4). In SMK, all individuals were homozygous for wild-type at all five amino acid positions. The typical *kdr* mutation, L1014F, was not detected in any of the Singapore populations and would not be expected in this species because *kdr* in *Aedes* would require a 2 nucleotides change.

**Table 4:** Transition of the frequency of the *Vssc* genotype during permethrin selection in the SP strain of *Aedes aegypti*.

Amino acid location						Percentage of mosquitoes (number of mosquitoes)				
989	1011	1014	1016	1534	1763	SMK (n = 24)	SPS_0_ (n = 48)	SPS_1_ (n = 48)	SPS_2_ (n = 24)	SPS_10_ (n = 24)
S/S	I/I	L/L	V/V	F/F	D/D	100 (24)	0	0	0	0
S/S	I/I	L/L	V/V	C/C	D/D	0	29.2 (14)	2.0 (1)	0	0
S/P	I/I	L/L	V/G	C/F	D/D	0	54.2 (26)	37.5 (18)	0	0
P/P	I/I	L/L	G/G	F/F	D/D	0	16.7 (8)	60.4 (29)	100 (24)	100 (24)
Frequencies of P989+G1016						0%	43.8%	79.2%	100%	100%

The subscript numbers in the population name indicate the number of generations exposed to permethrin. For example, SPS_2_ was the population which was selected by permethrin for two generations.

The location of amino acids are numbered according to the sequence of Vssc from the house fly, *Musca domestica* (accession nos. AAB47604 and AAB47605).

### Rate of permethrin reduction from cuticle

**Figure 2:**
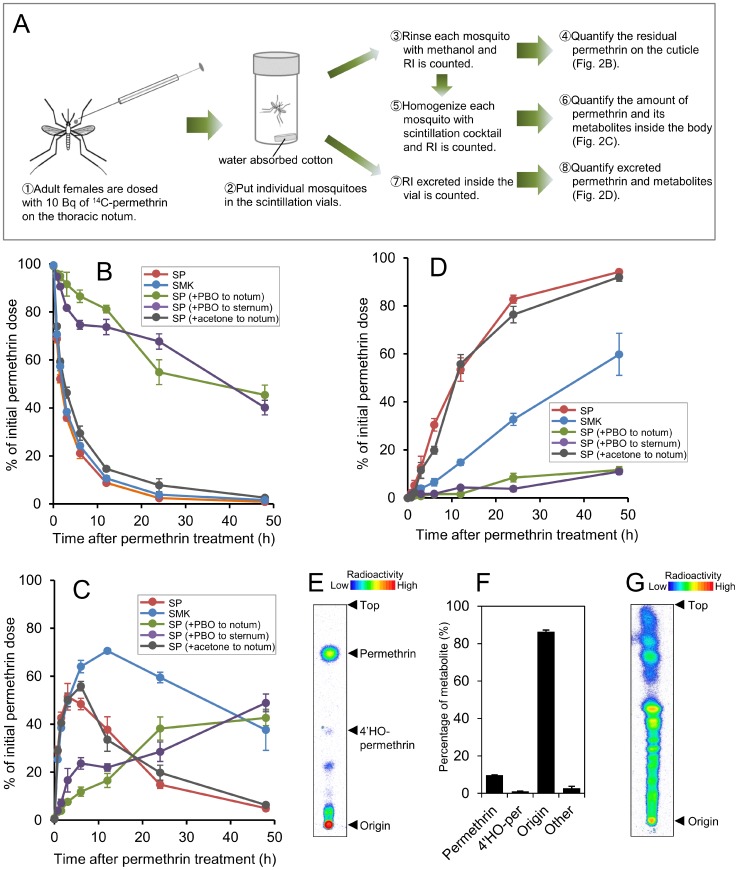
*In vivo* distribution of [^14^C]-permethrin topically applied to SP and SMK strains of *Aedes aegypti*. (A) Schematic of the procedure of *in vivo* assay. (B) Percentage of the applied radiolabel recovered in the external rinse. [^14^C]-Permethrin (600 dpm; 0.88 ng) was applied to the thoracic notum. PBO (5 µg) was also applied to the thoracic notum or thoracic sternum 1 h prior to permethrin application to observe its effect on permethrin penetration. Acetone was also applied 1 h prior to permethrin application to confirm whether the solvent had any side effects. Values are expressed as the means (±SE) for four mosquitoes. (C) Percentage of radioactivity detected in the mosquitoes. (D) Percentage of radioactivity excreted in the vials. (E) Thin-layer chromatogram of the compounds excreted by females of SP strain. Developing solvents: toluene/ethyl acetate (6∶1). The presence of 4′-HO-permethrin was determined by co-chromatography with an authenticated chemical. (F) Percentage of permethrin and its metabolites excreted by females of SP strain. Values represent the average and bars represent the SE of the mean (n  =  3). (G) Re-chromatography of the high-polar compounds stuck around origin. Developing solvents: chloroform/methanol/water (65∶25∶4).

We examined the rate of permethrin reduction from cuticle by measuring the disappearance of insecticide from the external surface of the mosquitoes (Figures 2A and B). There was no difference in the rate of permethrin reduction from cuticle between the SP and SMK strains, rather slightly higher in SP strain, with penetration rate constants of 0.358 h^−1^ and 0.252 h^−1^, respectively.

### Effects of PBO on permethrin penetration

Some previous studies suggested that synergistic effects of synergists are not due to inhibition of enzymes but due to enhancement of penetration rate of insecticides by synergists [52]–[54]. Therefore, we investigated the effects of PBO on permethrin penetration (Figure 2A). Pretreatment with PBO in the SP strain did not enhance permethrin penetration but resulted in significantly delayed its penetration (Figure 2B). Twelve hours after treatment, less than 20% of the insecticide had penetrated the cuticle in insects pretreated with PBO compared with 90% in insects without PBO treatment. A reduced rate of permethrin penetration was also observed even when we administered PBO to the thoracic sternum of mosquitoes. As shown in Figure 2B, pretreatment of the SP strain with acetone did not affect permethrin penetration.

### *In vivo* metabolism

The marked synergistic effects of PBO on permethrin toxicity implicated P450s being involved in resistance. Therefore we further investigated trends of permethrin after penetration through the cuticle using isotopic tracer tests. It was observed that in the SMK strain, internal radioactivity reached a peak at approximately 12 h after permethrin treatment, and then gradually decreased (Figure 2C). In contrast, in the SP strain, internal radioactivity reached a much earlier peak and was then rapidly eliminated. The percentage of the initial radioactivity that remained after 24 h in the SMK and SP strains was 59.5% and 14.8%, respectively. Unlike the groups without PBO pretreatment, the internal dose of radioactivity gradually increased in the groups that received PBO pretreatment and did not decrease until about 48 h after treatment. The internal radioactivity at 48 h with or without PBO in SP strain was 42.7% and 4.9%, respectively (Figure 2C).

Consistent with the above findings, we found that the SP strain excreted radioactivity more rapidly than the SMK strain (Figure 2D). The percentage of the radioactivity that was excreted 24 h after treatment in the SMK and SP strains was 32.7% and 82.8%, respectively. This suggested that the SP strain can eliminate permethrin more efficiently. The rate constants of excretion (hr^−1^) for SMK and SP were 0.022 ± 0.002 and 0.101 ± 0.008, respectively. PBO significantly reduced permethrin excretion, suggesting that permethrin is excreted after being metabolized by P450s. The high performance thin layer chromatography (HPTLC) analysis of excrete from SP strain revealed that more than 85% of the excreted radioisotope consisted of high polar compounds and approximately 10% of the permethrin was excreted without being metabolized (Figures 2E and F). Further investigation using another solvent specific for water-soluble metabolites showed that the compounds consisted of a number of metabolites (Figure 2G).

### *In vitro* metabolism

**Figure 3:**
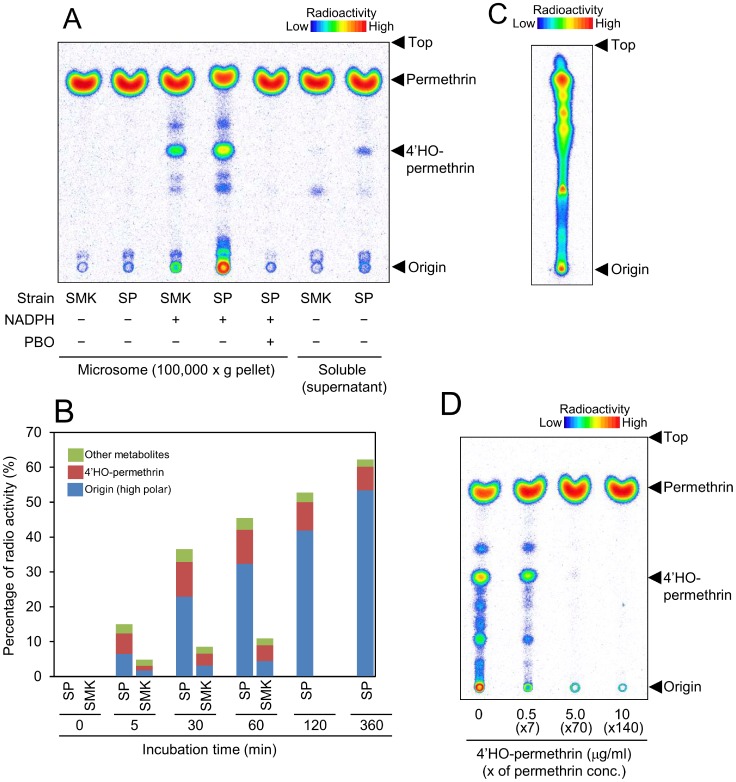
*In vitro* [^14^C]-permethrin metabolism studies. (A) Thin-layer chromatogram of *in vitro* permethrin metabolism in adult *Aedes aegypti* with or without NADPH or PBO. Microsomes (100,000 × *g* pellet) and the soluble fraction (100,000 × *g* supernatant) were used as the enzyme source. The presence of 4′-HO-permethrin was determined by co-chromatography with an authenticated chemical. (B) Metabolites of permethrin in adult *A. aegypti* at various incubation times. For detailed data, see Table S2. (C) Thin-layer chromatogram of high-polar compounds (SP) located at the origin of the first HPTLC. Developing solvents: chloroform/methanol/water (65∶25∶4). (D) Inhibition of permethrin metabolism by 4′-HO-permethrin. Microsomes of the SP strain were incubated for 30 min with [^14^C]-permethrin, NADPH, and ×7-, ×70-, or ×140-fold higher doses of 4′-HO-permethrin than permethrin.

We carried out *in vitro* metabolism studies to determine whether the changes observed *in vivo* were related to the activity of P450s. Microsomes of the SP strain metabolized permethrin at a much higher rate than those of the SMK strain (Figures 3A and B). Permethrin metabolism by microsomes was apparently due to P450 as it was NADPH-dependent and was inhibited by PBO (Figure 3A). Only a very small amount of permethrin was metabolized by enzymes in the 100,000 x*g* supernatant, suggesting that carboxyl esterase have only minimal involvement in the development of resistance. The rate constant for metabolism (min^−1^) of SMK and SP by microsomal enzymes was 0.0007 and 0.0042, respectively. At the start of incubation, 4′-HO-permethrin was the major metabolite in SP, although its percentage did not increase. In contrast, the percentage of high polar metabolites increased over time (Figure 3B, Table S2). The metabolites located around the origin of the HPTLC plates were collected and further developed with chloroform-based solvent (Figure 3C). The high-polar compounds consisted of a number of metabolites and were consistent with those in the *in vivo* study.

In order to assess the effect of 4′-HO-permethrin on permethrin metabolism, we performed an inhibition assay by incubating different doses of unlabeled 4′-HO-permethrin with [^14^C]-permethrin. This showed that unlabeled 4′-HO-permethrin inhibited effective permethrin metabolism, possibly by acting as a feedback inhibitor (Figure 3D).

### Microarrays

**Figure 4:**
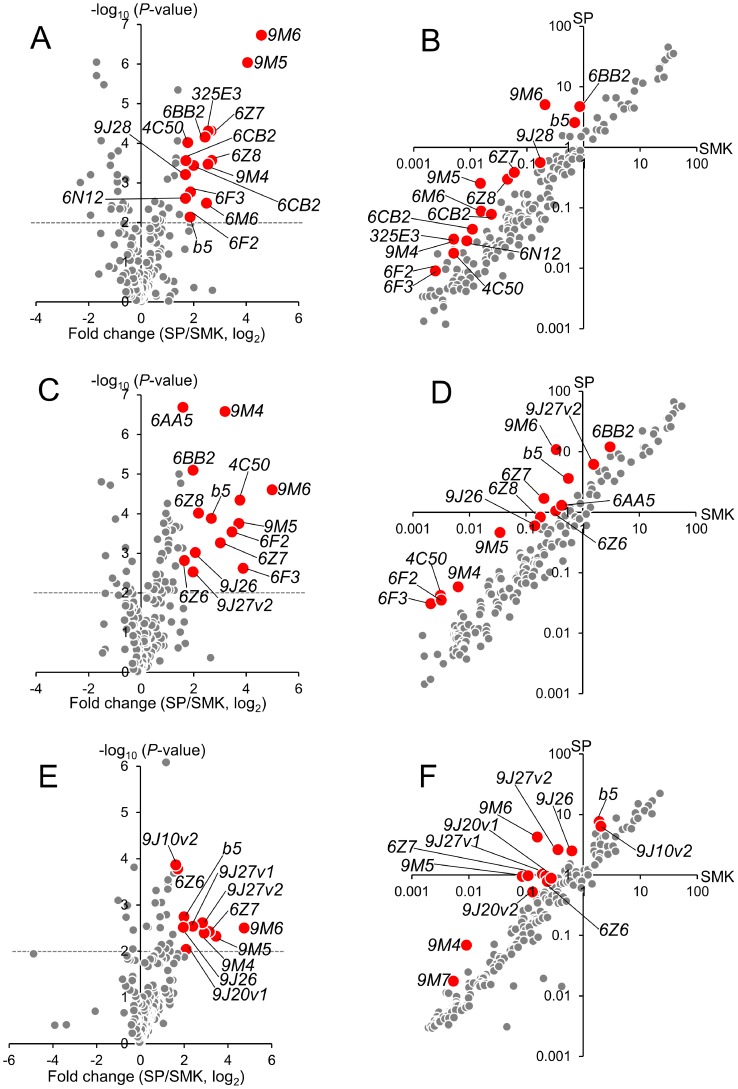
Microarray screening of cytochrome P450 and b_5_ genes differentially expressed in SP and SMK strains. Differential transcription of cytochrome P450 and b_5_ genes was individually investigated in adult female (A, B), adult male (C, D), and fourth instar larvae (E, F) of *Aedes aegypti*. Figures 4A–C show volcano plots of the relative changes (log_2_, x-axis) and statistical significance (–log_10_(*P*-value), y-axis). Figures 4D–F show scatter plots of the relative expression level of each P450 and b_5_ gene. Genes with relative expression >3-fold and a *P*-value<0.01 are highlighted.

A microarray that contained 60- mer probes for all genes identified by the genome project [55] was constructed. We then compared the levels of transcription in the SP and SMK strains of the adult males and females and the fourth instar larvae. In this study, we focused on P450s and their related genes because our investigations including bioassays and *in vivo* and *in vitro* metabolism studies showed that this metabolic enzyme is the key factor in the development of resistance. The genes over expressed in the SP strain were selected using a cut-off of >3-fold relative change in expression and a *P*-value<0.01 (Figure 4 and Table 5). Nine P450 genes (*CYP9M6*, *CYP9M5*, *CYP6Z8*, *CYP6Z7*, *CYP9M4*, *CYP6BB2*, *CYP6F3*, *CYP6F2*, and *CYP4C50*) were over expressed in both male and female insects. Out of nine, four (*CYP9M6*, *CYP9M5*, *CYP6Z7*, and *CYP9M4*) were over expressed in the larval sample as well. Cytochrome b_5_ was also over expressed in all three samples. *CYP9M6* was the only P450 that had a >20-fold transcription level with a significantly low (<0.01) *P*-value in all three samples. This gene had not been reported to be associated with insecticide resistance. Fifteen of the 22 P450 genes listed in Table 5 have previously been reported to be over expressed in one or more of pyrethroid-resistant strains/populations collected from Mexico [30], [56], Cuba [57], Thailand [30], Martinique, Bora Bora [58], and the Grand Cayman [57]. Two of these, *CYP9J26* and *CYP9J28* have been previously shown to have the ability to metabolize permethrin [59]. Other than P450s, no glutathione transferases (GSTs) or carboxyl esterases, except two GSTs, was over expressed in SP (under a cut off of >3-fold, *P*<0.01). GSTD4 (AAEL001054) was 11.9 (*P*  =  1.6 × 10^6^) and 7.9 (*P*  =  0.0025) times more expressed in SP of female and male, respectively, compared to SMK. GSTD5 (AAEL001071) in SP females was 5.5 (*P*  =  0.0020) times more expressed than in females of SMK.

**Table 5:** Cytochrome P450 and b_5_ genes differentially expressed in the SP (R) strain relative to the susceptible SMK (S) strain of *Aedes aegypti*.

		Female			Male			Larva			Strain or Population											
Gene name	Transcript ID	R/S		*P*	R/S		*P*	R/S		*P*	A	B	C	D	E	F	G	H	I	J	K	L
*CYP9M6*	AAEL001312	23.96	*	6.72	31.87	*	4.60	26.08	*	2.50												
*CYP9M5*	AAEL001288	16.64	*	6.03	13.25	*	3.75	10.97	*	2.32	**↑**											
*CYP6Z8*	AAEL009131	6.47	*	3.57	4.57	*	4.01	0.89		0.39		**↑**										
*CYP6Z7*	AAEL009130	6.36	*	4.31	8.11	*	3.26	8.88	*	2.42												
*CYP325E3*	AAEL000338	5.88	*	4.30	2.65		0.62	1.19		0.30												
*CYP9M4*	AAEL001320	5.86	*	3.47	9.18	*	6.58	7.55	*	2.39												
*CYP6M6*	AAEL009128	5.63	*	2.49	2.50		3.68	2.22		2.06			**↑**	**↑**								
*CYP6BB2*	AAEL014893	5.41	*	4.15	3.94	*	5.10	2.89		1.73	**↑**	**↑**	**↑**							**↑**		**↑**
*CYP6CB2*	AAEL002872	4.02	*	3.44	ND		ND	0.07		0.39								**↑**				
*CYP6F3*	AAEL014684	3.69	*	2.77	14.76	*	2.62	1.79		0.80												**↑**
*Cytochrome b_5_*	AAEL014935	3.65	*	2.14	6.42	*	3.88	3.98	*	2.74												
*CYP6F2*	AAEL014678	3.52	*	2.15	11.02	*	3.54	2.49		1.08												
*CYP4C50*	AAEL008017	3.44	*	4.02	13.63	*	4.34	2.07		0.44												
*CYP6CB2*	AAEL014924	3.27	*	3.56	3.21		0.73	1.80		0.89				**↑**				**↑**				
*CYP6N12*	AAEL009124	3.24	*	2.61	2.05		3.22	2.16		1.19	**↑**											
*CYP9J28*	AAEL014617	3.24	*	3.21	1.54		3.35	1.56		0.53	**↑**	**↑**				**↑**	**↑**	**↑**			**↑**	
*CYP9J26*	AAEL014609	3.52		1.78	4.20	*	3.02	3.91	*	2.51	**↑**	**↑**			**↑**							
*CYP9J27v2*	AAEL014607	3.69		1.96	3.95	*	2.53	7.19	*	2.62	**↑**				**↑**	**↑**						
*CYP6Z6*	AAEL009123	2.48		3.50	3.14	*	2.82	3.28	*	3.78				**↑**				**↑**	**↑**		**↑**	
*CYP6AA5*	AAEL012492	2.62		4.06	3.02	*	6.68	3.16		1.85			**↑**									
*CYP9J27v1*	AAEL014616	2.74		2.20	2.64		3.14	5.28	*	2.54	**↑**	**↑**			**↑**	**↑**						
*CYP9J20v1*	AAEL006814	1.08		0.09	1.93		4.01	4.27	*	2.04												
*CYP9J10v2*	AAEL014614	1.68		2.48	2.00		3.88	3.09	*	3.86	**↑**	**↑**				**↑**						

Asterisks indicate a relative change (R/S) >3-fold and a *P*-value (negative log_10_ scale) >2.

The symbol (**↑**) indicates over expression of the gene reported in the resistant population or the following strains:

A, Cayman (females) [57]; B, Cuba (females) [57]; C, Vauclin (larvae) [28]; D, Vauclin (males+females) [28]; E, PMDR (females) [30]; F, IM (males+females) [30]; G, IM (larvae) [30]; H, Iquitos (males+females) [56]; I, Calderitas (males+females) [56]; J, Lazaro C. (males+females) [56]; K, Merida (males+females) [56]; and L, Noexp-Perm (larvae) [58].

### Sequencing analysis of P450s

**Figure 5:**
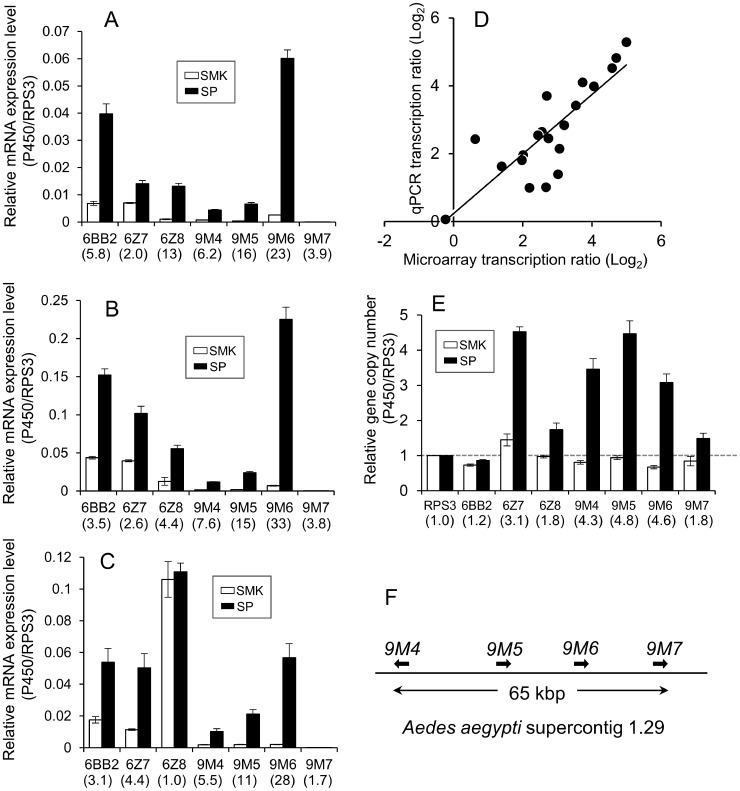
Real time quantitative PCR analysis of selected genes from the microarray experiments. Transcription levels of seven P450 genes were individually validated in adult female (A), adult male (B), and fourth instar larvae (C) in SP and SMK strains of *Aedes aegypti*. Ribosomal protein S3 gene (*RPS3*) was used to normalize the data. Expression ratios (SP/SMK) are expressed in parentheses. (D) Correlation between the microarray and real time quantitative PCR (R^2^  =  0.824). (E) Gene copy number of 7 P450s and *RPS3* quantified by real time PCR. The ratios of relative copy number (SP/SMK) are expressed in parentheses. (F) Gene clusters of cytochrome P450 and the CYP9M subfamily on the *A. aegypti* supercontig 1.29 on the 2nd chromosome. The error bars represent standard errors of three (A, B, and C) and eight (E) biological replicates.

In order to design primers for real time quantitative PCR, we used eight mosquitoes individually to obtain the sequence of partial or full length DNA for seven P450 genes (*CYP6BB2*, *CYP6Z7*, *CYP6Z8*, *CYP9M4*, *CYP9M5*, *CYP9M6* and *CYP9M7*) and an internal control gene, ribosomal protein S3 (*RPS3*). We focused on these P450 genes according to the following criteria: overexpressed in females (>5) and in males (>3) in SP relative to SMK in microarray analysis. *CYP9M7* was also analyzed as it forms a gene cluster with *CYP9M4*, *CYP9M5*, and *CYP9M6* within 65 kbp on the same supercontig (1.29) and is structurally related to these genes (Figure 5F). The full length cDNA and deduced amino acid sequences of *CYP6BB2* were identical between the SP and SMK strains. However, on the ninth nucleotide after the stop codon, we observed a C1533T replacement in the SP strain (Figure S3). All eight SP mosquitoes possessed two *CYP9M6* genes, designated as *CYP9M6v1* (accession number AB840269) and *CYP9M6v2* (accession number AB840270) (Figures S3 and S4). The nucleotide and amino acid identities of these alleles were 97.6% (1563/1602) and 98.9% (527/533), respectively. In the SMK strain, we found three *CYP9M6* variants; two mosquitoes were homozygous for *CYP9M6v3* (accession number AB846835) or *CYP9M6v4* (accession number AB846836), two were homozygous for *CYP9M6v5* (accession number AB846837), and four were heterozygous *CYP9M6* variants. The CYP9M6v5 protein appeared to be incomplete as an active enzyme because there was a stop codon at amino acid position 473 (Figure S4).

### Quantitative real-time PCR

We performed real-time quantitative PCR (qPCR) for seven P450 genes to verify the results of the microarray analyses. We focused these genes according to the criteria described above. Primers for quantitative real time PCR were designed from sequences that were consensus among eight SP and SMK mosquitoes. Five of these genes, *CYP6BB2*, *CYP6Z7*, *CYP9M4*, *CYP9M5*, and *CYP9M6* were over expressed during the larval stage and in adult males and females (Figures 5A–C) in SP relative to SMK. As shown in Figure 5D there was a good correlation between the results of the microarrays and qPCR (R^2^  =  0.824). This confirmed that the results of the microarray were accurate. *CYP9M6* showed the largest relative change in the seven genes and was over expressed 22.9-fold in males, 38.9-fold in females, and 28.2-fold in larvae of the SP strain relative to SMK. The relative expression level of *CYP6BB2* to *RPS3* was also higher (close to *CYP9M6*), especially in adult females (Figure 5A), with the gene reported to be over expressed in five other pyrethroid-resistant strains (Table 5).

### Identification of the gene copy number

The gene copy numbers of the seven P450s in the SP and SMK strains were compared by qPCR (Figure 5E). *RPS3* gene was used to normalize the data. While the majority of the P450 genes in the SMK strain were likely to be a single copy, some genes in SP were amplified significantly. The average copy number of *CYP6Z7*, *CYP9M4*, *CYP9M5*, and *CYP9M6* in SP were 3.1-, 4.3-, 4.8-, and 4.6-fold more than SMK, respectively. This clearly shows that the over expression of these P450 genes, at least in part, was due to gene amplification.

### Association between permethrin excretion and *CYP9M6* and *CYP6BB2*

**Figure 6:**
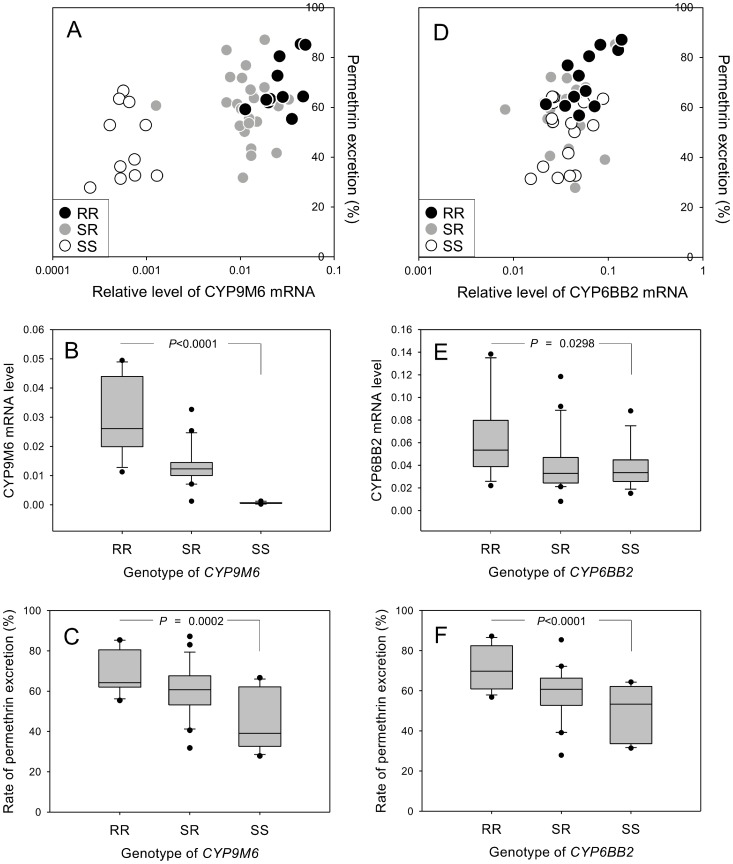
Genotype–gene expression–phenotype associations. The association between *CYP9M6*/*CYP6BB2* genotypes and mRNA expression and permethrin excretion rate was investigated. The female F2 populations of SP and SMK strains were dosed with [^14^C]-permethrin, and the amount of permethrin excreted was individually quantified 24 h after treatment. The genotype of each mosquito was identified by the genomic DNA isolated from six legs. mRNA level was also quantified with RNA isolated from the body part of each mosquito. For more information, see Materials and Methods. (A, D) Plot showing the association between the level of mRNA and permethrin excretion in *CYP9M6* (A) and *CYP6BB2* (D). (B, E) Box plot showing the association between mRNA level and *CYP9M6* (B) and *CYP6BB2* (E). (C, F) Box plot showing the association between permethrin excretion rate and genotype of *CYP9M6* (C) and *CYP6BB2* (F). *P*-values for comparison of the RR and SS genotypes were calculated using Dunnett's test.

In order to determine whether CYP9M6 and CYP6BB2 have a role in permethrin metabolism, F2 progeny (interbred population of F1 (SMK × SP)) were treated with a sub-lethal dose of [^14^C]-permethrin, and the excretion rate was measured 24 h after treatment in individuals. The genotypes and transcription levels of *CYP9M6* and *CYP6BB2* were determined for each mosquito to examine the association between these parameters and the rate of excretion (Figures 6A and D). The transcription levels for *CYP9M6* between RR (homozygote of SP allele) and SS (homozygote of SMK allele) were significantly different (*P*<0.0001), with a 45-fold difference between the two genotypes (Figure 6B). The rate of permethrin excretion was 69% for RR and 45% for SS (Figure 6C). On the other hand, although the transcription level of *CYP6BB2* was significantly different between RR and SS (*P*  =  0.0298), the relative change (RR/SS) was only 1.6-fold (Figure 6E). The rate of permethrin excretion in RR (71%) was significant than that in SS (49%) (P<0.0001, Figure 6F). These results suggested that both CYP9M6 and CYP6BB2 were associated with rapid excretion of permethrin, although it was likely that the metabolism rate of permethrin was different between the two isozymes.

**Figure 7:**
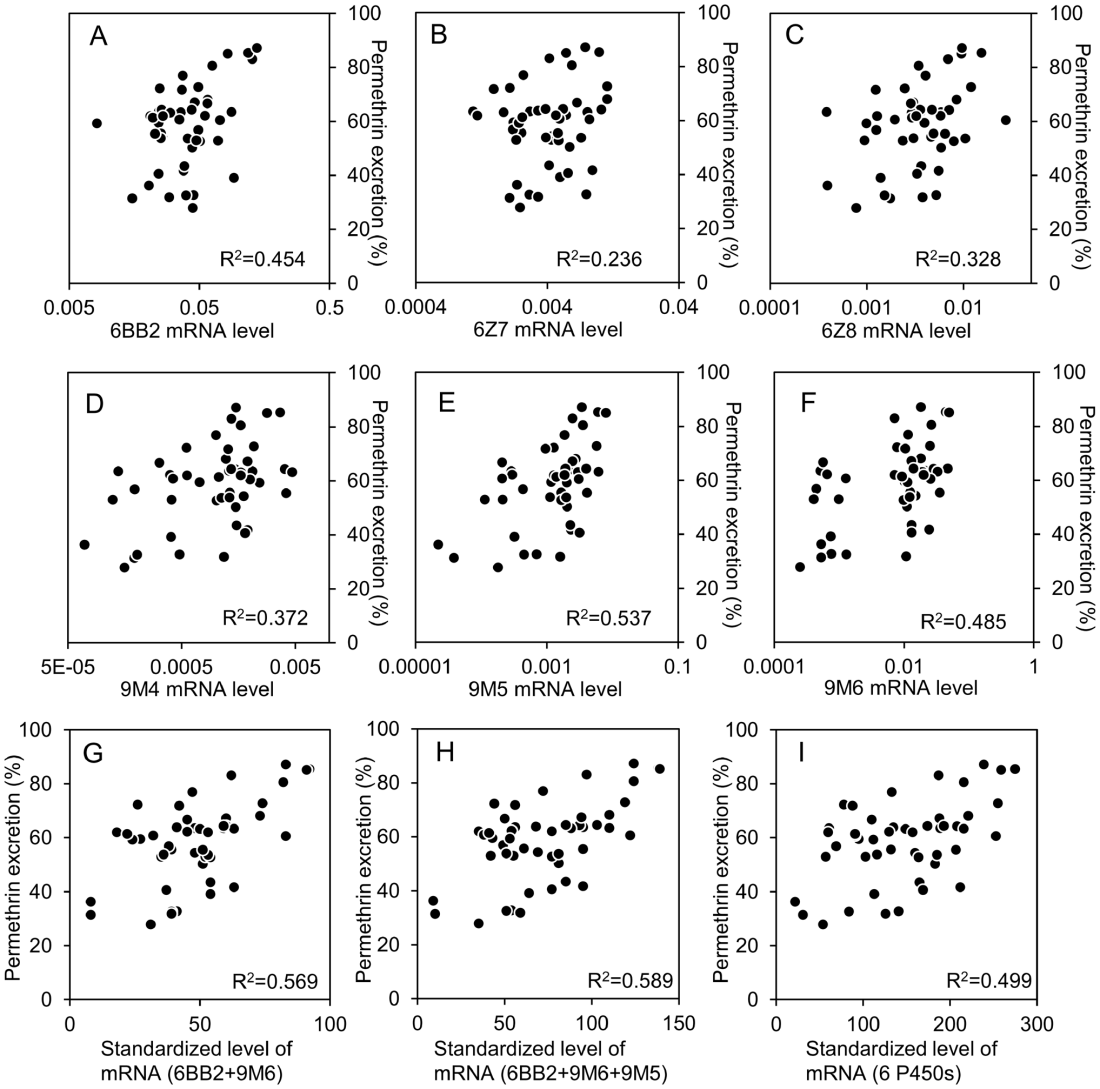
Associations between the gene expression level of six P450s and permethrin excretion rate. The female F2 populations of SP and SMK strains were dosed with [^14^C]-permethrin and the amount of permethrin excreted by each insect was quantified 24 h after treatment. The level of mRNA was quantified using RNA isolated from each mosquito. For more information, see Materials and Methods. (A) *CYP6BB2*, (B) *CYP6Z7*, (C) *CYP6Z8*, (D) *CYP9M4*, (E) *CYP9M5*, and (F) *CYP9M6*. Forty-eight individuals were ranked from 1 to 48 according to the level of expression of each P450 gene. The ranks against multiple P450 genes were combined to standardize the level of mRNA and evaluate the association between permethrin excretion rate and expression level of multiple P450 genes. (G) Association between permethrin excretion and standardized level of mRNA for CYP6BB2 and CYP9M6. (H) Association between permethrin excretion rate and standardized level of mRNA for CYP6BB2, CYP9M6 and CYP9M5. (I) Association between permethrin excretion rate and standardized level of mRNA for CYP6BB2, CYP6Z7, CYP6Z8, CYP9M4, CYP9M5, and CYP9M6.

In addition to CYP6BB2 and CYP9M6, the transcription levels of other four P450 genes (CYP6Z7, CYP6Z8, CYP9M4, and CYP9M5) were quantified in 48 F2 progeny in order to determine their association with the rate of permethrin excretion (Figure 7). We observed a relatively high correlation between the rate of permethrin excretion and mRNA levels for CYP6BB2 and CYP9M6, with correlation coefficients (R^2^) of 0.454 and 0.485, respectively (Figures 7A and F). The R^2^ for CYP9M5 was also high (0.537, Figure 7E). Of the six P450 genes, CYP6Z7 had the lowest R^2^ (0.236, Figure 7B). Forty-eight individuals were ranked from 1 to 48 according to the level of expression of each P450 gene. The ranks of each individual against multiple P450 genes were combined to standardize the level of mRNA and we evaluated the association between permethrin excretion rate and expression level of multiple P450 genes. The standardized transcription levels of CYP9M6+CYP6BB2 had a considerably higher R^2^ (0.569) than individual values alone (Figure 7G). This strongly suggests that both these P450s contribute to permethrin excretion. The combination of CYP9M6, CYP6BB2, and CYP9M5 further increased the R^2^ to 0.589 (Figure 7H), whereas the combination of all six P450s decreased the R^2^ to 0.499 (Figure 7I). These findings imply that not all of these P450 were involved in permethrin excretion.

### Metabolism of permethrin by heterologously expressed CYP9M6 and CYP6BB2

**Figure 8:**
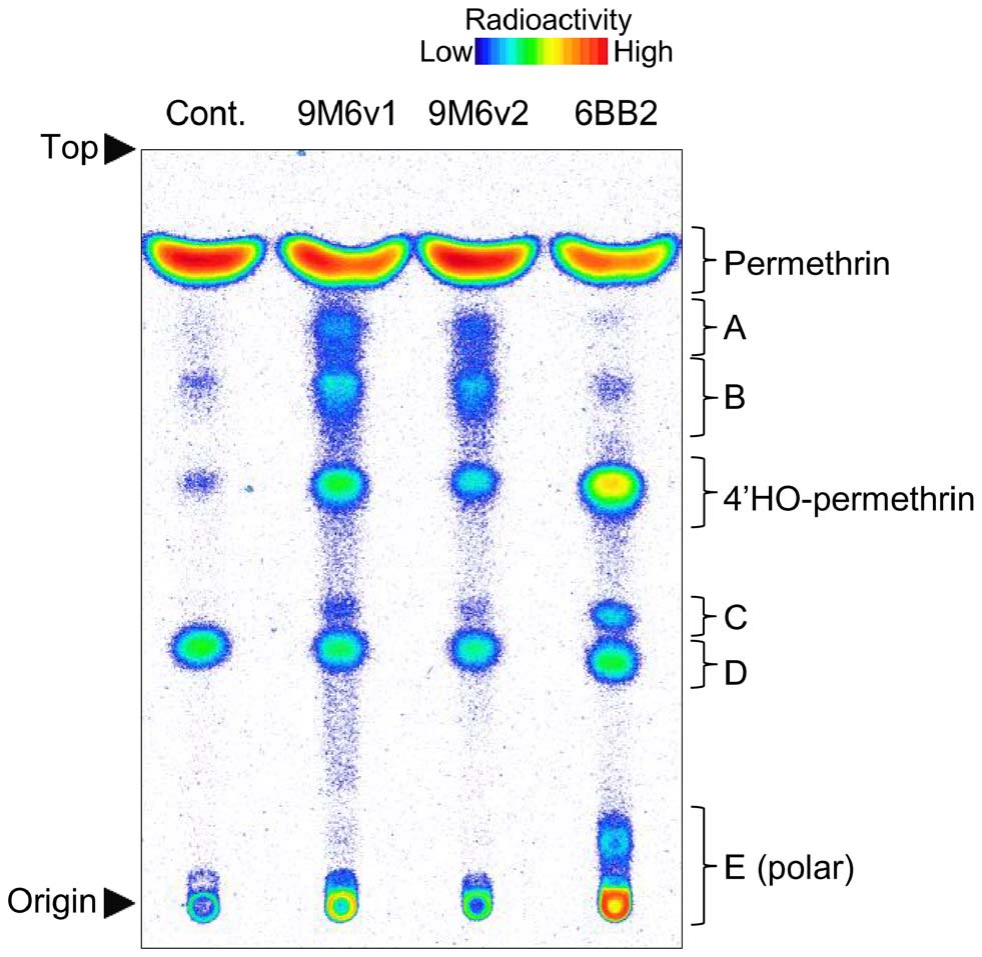
Thin layer chromatogram of [^14^C]-permethrin metabolites by heterologously expressed CYP9M6v1, CYP9M6v2, and CYP6BB2. A-E denote unidentified metabolites. Developing solvents: toluene/ethyl acetate (6∶1). The presence of 4′-HO-permethrin was determined by co-chromatography with an authenticated chemical.

In order to determine whether CYP9M6 and CYP6BB2 are capable of permethrin metabolism, these P450s were co-expressed with *A. aegypti* cytochrome P450 reductase and b_5_ in Sf9 cells using a baculovirus. For *CYP9M6*, two genes, *CYP9M6v1* and *CYP9M6v2* were expressed, as sequencing analysis showed that all individuals in the SP strain possessed both types of genes. Microsomes were prepared from cells infected with baculovirus, followed by an *in vitro* metabolism study using [^14^C]-permethrin as the substrate. Both CYP9M6v1 and CYP9M6v2 demonstrated relatively low but consistent permethrin metabolism, whereas CYP6BB2 exhibited strong metabolic activity for permethrin (Figure 8, Table S3). The compound, 4′-HO-permethrin, detected in the microsome metabolism studies, was also confirmed as a major metabolite for the three P450 samples (CYP9M6v1, CYP9M6v2, and CYP6BB2). This suggested that these three P450s can detoxify permethrin. In addition, a large amount of polar metabolites (E) were detected by *in vivo* and *in vitro* studies on all three samples. Unique metabolites (metabolites A and B), which were almost undetectable in the *in vitro* microsomal study were also detected in samples of CYP9M6v1 and CYP9M6v2 (Figure 8, Table S3).

## Discussion

We selected a Singapore colony of *A. aegypti* (SPS_0_) in the laboratory and established a SP ( = SPS_10_) strain, which developed an extremely high level of resistance to permethrin. Using bioassays with a synergist and *in vivo* and *in vitro* studies, we confirmed that P450s play very important roles in the development of resistance. The larvae of the SP strain also developed a high level of resistance despite the selections being conducted on adult mosquitoes. This indicates that common mechanism(s) causes resistance in both developmental stages. This finding is in contrast to those reported in the JPal-per strain (*Culex quinquefasciatus*), with these insects being selected by permethrin in the larval stage and not showing a high level of resistance in the adult stage [60]. The JPal-per strain has a knockdown resistance gene allele (L1014F) as well plus high expression levels of *CYP9M10*, known to confer resistance [61]–[63], being approximately 250-fold higher than in the larvae of susceptible strain, and showing limited expression in the adult stage [37]. In the SP strain, *CYP9M6*, *CYP6BB2*, and *CYP9J26* were over expressed in both the larval and adult stages and probably these were involved in the larval resistance as well.

The amino acid substitutions of Vssc, V1016G and F1534C, have been shown to strongly correlate with pyrethroid resistance in *A. aegypti* [17], [23]–[27]. The Singapore population (SPS_0_) possessed both G1016 and C1534, with a gene frequency of 44% and 56%, respectively. However, all C1534 haplotypes were disappeared after only two selections by permethrin. This clearly indicates that Vssc with a G1016 mutation has a lower sensitivity to permethrin than channel with a C1534 mutation. This finding is consistent with that reported by a recent neurophysiological study [27]. The results of our bioassays further supported this phenomenon, as we showed 3–5-fold differences in resistance ratios between SP and SPS_0_ insects when they were treated permethrin with PBO in the adult (Table 2) and also larval stages (Table 3). Changes of *Vssc* genotype is also associated with development of resistance in the larval stage in SP. The reason for relatively high frequency of C1534 mutation in this country is still unclear. Further studies, including the examination of fitness costs between G1016 and C1534, will help us better understand factors affect the equilibrium state of these two haplotypes in nature.

In the bioassays, PBO exhibited a marked synergistic effect on the toxicity of permethrin in the SP strain (Table 2 and 3). On the other hand, several research groups stated that synergists may enhance the toxicity of insecticides because they accelerate the penetration of these agents [52]–[54]. We therefore examined the effects of PBO on permethrin penetration and showed that it did not enhance permethrin penetration, but instead suppressed the rate of its penetration (Figure 2). This finding is consistent with other reports on the German cockroach [64] and house fly [65]–[67]. We also found that permethrin penetration was markedly suppressed by PBO treatment, even administered on the thoracic sternum of the insect followed by permethrin treatment on the thoracic notum. This result implies that PBO was not acting as a physical barrier to permethrin penetration but rather another mechanism. We speculate that high concentration of PBO inside the mosquito body suppressed permethrin passing through the cuticle by the effect of a concentration gradient of chemicals. Therefore, caution must be exercised when we use a synergist in a bioassay to avoid underestimating the contribution of metabolic enzymes to the resistance.

**Figure 9:**
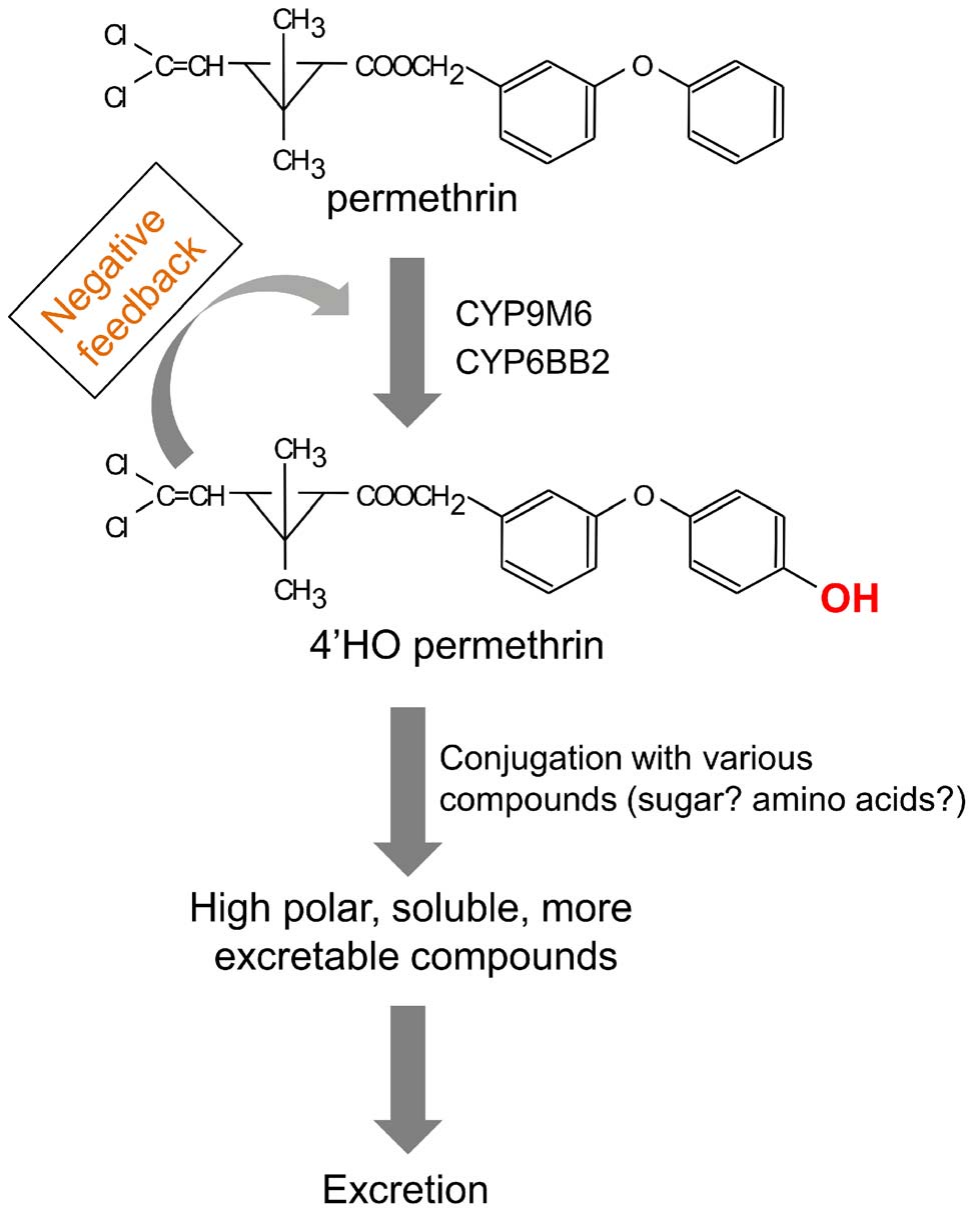
Proposed model showing the metabolic pathway of permethrin.

4′-HO-permethrin has been well documented as a primary metabolite of permethrin in various insects including the house fly, southern house mosquito, cockroach, and cabbage looper [42], [44], [68]. We also showed that this compound was a major metabolite of permethrin in the SP strain *in vitro* and comprised approximately 40% of the metabolites after an incubation of 5 min (Figure 3B). However, the rate of 4′-HO-permethrin to the total metabolites decreased over time and comprised approximately 10% after 6 h. On the other hand, the rate of high polar compounds increased over time and made up 86% of the total metabolites after 6 h. Further, 4′-HO-permethrin was barely detectable in the metabolites excreted by mosquitoes *in vivo* (Figures 2E and F). This suggested that 4′-HO-permethrin was further metabolized to secondary metabolites that were more water soluble and excretable (Figure 9). We also found that 4′-HO-permethrin inhibited permethrin metabolism *in vitro* (Figure 3D), indicating the presence of a negative feedback regulation (Figure 9). Insects need to convert hydroxyl-permethrin to other secondary metabolites in order to avert this negative feedback, resulting in insufficient excretion. Secondary development in the HPTLC analysis showed that the high polar metabolite consisted of a number of various compounds (Figures 2G and 3C). These compounds were suspected of being conjugates of 4′-HO-permethrin with glucosides or/and with various amino acids including glycine, glutamic acid, glutamine, and serine, as reported by Shono *et al*. [68]. It remains unknown what enzymes play the role in the secondary metabolism of pyrethroid in insects, and it is possible that an obscure mechanism of insecticide resistance may exist.

Association of gene amplification with insecticide resistance is relatively well documented [69]. In organophosphorus-resistant aphids and *Culex* mosquitoes, genes of carboxyl esterases are amplified 80–250 times and the capability to detoxify insecticides is increased [70]–[72]. Recent studies demonstrated that gene amplification is also associated with over expression of P450 genes in insects [40], [62], [63], [73], [74]. In *A. aegypti*, over expression of *CYP9J29* in the CUBA-DELTA and CAYMAN strains was shown to be due to gene amplification [57]. In the current study, we found that CYP9M6 had the capability to metabolize permethrin and was over expressed in the SP strain partially due to gene amplification. Real time quantitative PCR revealed that this gene was amplified approximately 4-fold in the SP strain compared with the SMK strain. We identified two *CYP9M6* genes, *CYP9M6v1* and *CYP9M6v2*, which were probably raised by the gene amplification event in the SP strain. In particular, two other similar genes, *CYP9M4* and *CYP9M5*, located within the same cluster on chromosome two [75] were also amplified to a similar extent (Figure 5E). This suggested that this amplification may have occurred coincidently.

Our microarray analysis revealed that 22 P450 genes were over expressed >3-fold in all samples (males, females, or larvae) of the resistant SP strain compared with the susceptible SMK strain (Table 5). It is noteworthy that 15 of these genes have been reported previously to be overexpressed in one or more resistant populations or strains collected from different regions of the world [28], [30], [56], [57]. In this study, we found that CYP9M6 and CYP6BB2 were responsible for resistance. In addition, *CYP9J26* and *CYP9J28*, which have the ability to metabolize permethrin [59], [76], were also over expressed in the SP strain. It remains to be determined as to how many P450s contribute to the resistance in SP mosquitoes. However, it is not likely that all genes over expressed in the SP strain are involved in the development of resistance, as it is generally known that transcription of genes forming clusters are occasionally coordinately controlled by the same regulatory factor [77]. In association analysis using F2 progeny, the expression level of *CYP9M5* and *CYP9M6* showed a high correlation coefficient (R^2^  =  0.90), whereas no correlation was observed between the expression levels of *CYP9M6* and *CYP6BB2* (R^2^  =  0.10, data not shown). This suggested that *CYP9M5* and *CYP9M6* are controlled partially by a common mechanism. A further example of this mechanism is the finding that administration of phenobarbital to insects induces a multiple number of P450 genes [78], [79]. Therefore, over expressed genes do not always provide an advantage for insect survival. Heterologous expression and confirmation of the metabolic activity of each P450 isoform is necessary to gain a greater understanding of these processes.

To the best of our knowledge, this is the first report showing a correlation between the expression level of each P450 and the rate of insecticide metabolism of individual insects. This analysis may provide a good tool for evaluating the rate of contribution of each P450 isoform to the development of resistance. In this analysis, both *CYP9M6* and *CYP6BB2* showed moderate correlation between the levels of transcription and the individual rate of permethrin metabolism. Furthermore, although no correlation was observed between the expression level of *CYP9M6* and *CYP6BB2* (R^2^  =  0.10), there was a prominent relationship between the rate of permethrin metabolism and standardized-combined expression level of *CYP9M6* and *CYP6BB2* (Figure 7G). These results strongly suggest that these two P450s are auxiliary parts of effective permethrin excretion. Although *CYP9M6* showed the greatest relative change in gene expression in the comparison between the SP and susceptible strains, we still hesitate to conclude that this enzyme plays a major role in permethrin metabolism. First, homozygotes of *CYP6BB2* had a rate of permethrin excretion as high as that observed in homozygotes of *CYP9M6* in Figures 6C and F. Second, in the heterologous expression study, under our experimental conditions, CYP6BB2 showed a much higher activity of permethrin metabolism than CYP9M6. It is therefore possible that CYP9M6 may compensate for its low metabolic activity by its high mRNA level. Although both *CYP9M4* and *CYP9M5* were also over expressed in the SP strain, we did not investigate the permethrin metabolic activity of these P450s because their relative expression level was below 1/10^th^ of that of *CYP9M6*. However, it is possible they may confer resistance depending on their unit activity of permethrin metabolism. Further work will be needed to clarify if these P450 isoforms are involved in the resistance.

One of the goals of the study was to establish a more accurate molecular diagnosis method for monitoring field populations of *A. aegypti* by identifying metabolic enzymes that confer resistance. Findings of CYP9M6 and CYP6BB2 are good progress for this purpose, however, we also found that the high level of resistance in the SP strain was not due to one major mechanism but was actually the consequence of multiple P450 isozymes (at least 4) and reduced sensitivity of Vssc. This implies that we may need to recognize the reality that the development of molecular diagnoses targeting metabolic enzymes will be more complicated and challenging than that of targeting only *Vssc* [80]. We are currently focusing on elucidating the contribution degree of these P450s on pyrethroid resistance in the field populations of *A. aegypti* collected from different regions.

## Supporting Information

Figure S1 Diagram depicting primer positions.
(PDF)

Figure S2 Multiple alignments of *CYP9M6* cDNA sequences identified from SP and SMK strains of *Aedes aegypti*. Hyphens indicate identical nucleotides to the sequence of *CYP9M6v1* (SP). *CYP9M6v1* and *v2* are identified from SP strain. *CYP9M6v3*, *v4*, and *v5* were identified from SMK strain. Primers used for genotyping were also indicated. For the deduced amino acid sequences, see Figure S4.
(PDF)

Figure S3 Full length cDNA and deduced amino acid sequence of CYP6BB2 from SP and SMK strains of *Aedes aegypti*. The red letter indicates the only nucleotide differentiate between SP (cytosine) and SMK (thymidine) in this region. The 6BB2F21 and 6BB2R22 primers were used for genotyping. The poly(A) addition signal (aataaa) is also underlined.
(PDF)

Figure S4 Multiple alignments of CYP9M6 amino acid sequences identified from Liverpool, SP, and SMK strains of *Aedes aegypti*. Hyphens indicate identical amino acids to the sequence of Liverpool strain which was used for the genome project. Asterisks indicate stop codons.
(PDF)

Table S1 Primer lists.
(XLSX)

Table S2 *In vitro* metabolism of [^14^C]-permethrin by microsome of SP and SMK strains of *Aedes aegypti*.
(PDF)

Table S3 Metabolism of [^14^C]-permethrin by CYP9M6v1, CYP9M6v2, and CYP6BB2 expressed in Sf9 cells.
(PDF)

## References

[1] Mackenzie JS, Gubler DJ, Petersen LR (2004) Emerging flaviviruses: the spread and resurgence of Japanese encephalitis, West Nile and dengue viruses. Nat Med 10: S98–S109. 15577938 10.1038/nm1144

[2] Gubler DJ (2002) Epidemic dengue/dengue hemorrhagic fever as a public health, social and economic problem in the 21st century. Trends Microbiol 10: 100–103. 11827812 10.1016/s0966-842x(01)02288-0

[3] Gubler DJ, Clark GG (1995) Dengue/dengue hemorrhagic fever: the emergence of a global health problem. Emerg Infect Dis 1: 55–57. 8903160 10.3201/eid0102.952004 PMC2626838

[4] Whitehorn J, Farrar J (2010) Dengue. Br Med Bull 95: 161–173. 20616106 10.1093/bmb/ldq019

[5] Gubler DJ (1998) Dengue and dengue hemorrhagic fever. Clin Microbiol Rev 11: 480–496. 9665979 10.1128/cmr.11.3.480 PMC88892

[6] Hemingway J, Ranson H (2000) Insecticide resistance in insect vectors of human diseases. Annu Rev Entomol 45: 371–391. 10761582 10.1146/annurev.ento.45.1.371

[7] Hemingway J, Hawfes NJ, McCarroll L, Ranson H (2004) The molecular basis of insecticide resistance in mosquitoes. Insect Biochem Mol Biol 34: 653–665. 15242706 10.1016/j.ibmb.2004.03.018

[8] Vontas J, Kioulos E, Pavlidi N, Morou E, della Torre A, et al (2012) Insecticide resistance in the major dengue vectors *Aedes albopictus* and *Aedes aegypti* . Pestic Biochem Physiol 104: 126–131.

[9] Goh KT (1997) Dengue-a re-emerging infectious disease in Singapore. Ann Acad Med Singapore 26: 664–670. 9494676

[10] Ooi EE, Goh KT, Gubler DJ (2006) Dengue prevention and 35 years of vector control in Singapore. Emerg Infect Dis 12: 887–893. 16707042 10.3201/10.3201/eid1206.051210 PMC3373041

[11] Lee KS, Lai YL, Lo S, Barkham T, Aw P, et al (2010) Dengue virus surveillance for early warning, Singapore. Emerg Infect Dis 16: 847–849. 20409381 10.3201/eid1605.091006 PMC2953985

[12] Koh BKW, Ng LC, Kita Y, Tang CS, Ang LW, et al (2008) The 2005 dengue epidemic in Singapore: epidemiology, prevention and control. Ann Acad Med Singapore 37: 538–545. 18695764

[13] Koou SY, Chong CS, Vythilingam I, Ng LC, Lee CY (2014) Pyrethroid resistance in *Aedes aegypti* larvae (Diptera: Culicidae) from Singapre. J Med Entomol 51: 170–181. 24605467 10.1603/me13113

[14] Rinkevich FD, Du Y, Dong K (2013) Diversity and convergence of sodium channel mutations involved in resistance to pyrethroids. Pestic Biochem Physiol 106: 93–100. 24019556 10.1016/j.pestbp.2013.02.007 PMC3765034

[15] Shono T (1985) Pyrethroid resistance: importance of the *kdr*-type mechanism. J Pestic Sci 10: 141–146.

[16] Srisawat R, Komalamisra N, Eshita Y, Zheng M, Ono K, et al (2010) Point mutations in domain II of the voltage-gated sodium channel gene in deltamethrin-resistant *Aedes aegypti* (Diptera:Culicidae). Appl Entomol Zool 45: 275–282.

[17] Saavedra-Rodriguez K, Urdaneta-Marquez L, Rajatileka S, Moulton M, Florest AE, et al (2007) A mutation in the voltage-gated sodium channel gene associated with pyrethroid resistance in Latin American *Aedes aegypti* . Insect Mol Biol 16: 785–798. 18093007 10.1111/j.1365-2583.2007.00774.x

[18] Rajatileka S, Black IV WC, Saavedra-Rodriguez K, Trongtokit Y, Apiwathnasorn C, et al (2008) Development and application of a simple colorimetric assay reveals widespread distribution of sodium channel mutations in Thai populations of *Aedes aegypti* . Acta Trop 108: 54–57. 18801327 10.1016/j.actatropica.2008.08.004

[19] Chang C, Shen WK, Wang TT, Lin YH, Hsu EL, et al (2009) A novel amino acid substitution in a voltage-gated sodium channel is associated with knockdown resistance to permethrin in *Aedes aegypti* . Insect Biochem Mol Biol 39: 272–278. 19171193 10.1016/j.ibmb.2009.01.001

[20] Yanola J, Somboon P, Walton C, Nachaiwieng W (2010) A novel F1552/C1552 point mutation in the *Aedes aegypti* voltage-gated sodium channel gene associated with permethrin resistance. Pestic Biochem Physiol 96: 127–131.

[21] García GP, Flores AE, Fernández-Salas I, Saavedra-Rodríguez K, Reyes-Solis G, et al (2009) Recent rapid rise of a permethrin knock down resistance allele in *Aedes aegypti* in México. PLoS Negl Trop Dis 3: e531. 19829709 10.1371/journal.pntd.0000531 PMC2759509

[22] Kawada H, Higa Y, Komagata O, Kasai S, Tomita T, et al (2009) Widespread distribution of a newly found point mutation in voltage-gated sodium channel in pyrethroid-resistant *Aedes aegypti* populations in Vietnam. PLoS Negl Trop Dis 3: e527. 19806205 10.1371/journal.pntd.0000527 PMC2754656

[23] Harris AF, Rajatileka S, Ranson H (2010) Pyrethroid resistance in *Aedes aegypti* from Grand Cayman. Am J Trop Med Hyg 83: 277–284. 20682868 10.4269/ajtmh.2010.09-0623 PMC2911171

[24] Saavedra-Rodriguez K, Strode C, Suarez AF, Salas IF, Ranson H, et al (2008) Quantitative trait loci mapping of genome regions controlling permethrin resistance in the mosquito *Aedes aegypti* . Genetics 180: 1137–1152. 18723882 10.1534/genetics.108.087924 PMC2567363

[25] Brengues C, Hawkes NJ, Chandre F, McCarroll L, Duchon S, et al (2003) Pyrethroid and DDT cross-resistance in *Aedes aegypti* is correlated with novel mutations in the voltage-gated sodium channel gene. Med Vet Entomol 17: 87–94. 12680930 10.1046/j.1365-2915.2003.00412.x

[26] Hu Z, Du Y, Nomura Y, Dong K (2011) A sodium channel mutation identified in *Aedes aegypti* selectively reduces cockroach sodium channel sensitivity to type I, but not type II pyrethroids. Insect Biochem Mol Biol 41: 9–13. 20869441 10.1016/j.ibmb.2010.09.005 PMC3022105

[27] Du Y, Nomura Y, Satar G, Hu Z, Nauen R, et al (2013) Molecular evidence for dual pyrethroid-receptor sites on a mosquito sodium channel. PNAS 110: 11785–11790. 23821746 10.1073/pnas.1305118110 PMC3718148

[28] Marcombe S, Poupardin R, Darriet F, Reynaud S, Bonnet J, et al (2009) Exploring the molecular basis of insecticide resistance in the dengue vector *Aedes aegypti*: a case study in Martinique Island (French West Indies). BMC Genomics 10: 494. 19857255 10.1186/1471-2164-10-494 PMC2770535

[29] Scott JG (1999) Cytochrome P450 and insecticide resistance. Insect Biochem Mol Biol 29: 757–777. 10510498 10.1016/s0965-1748(99)00038-7

[30] Strode C, Wondji CS, David JP, Hawkes NJ, Lumjuan N, et al (2008) Genomic analysis of detoxification genes in the mosquito *Aedes aegypti* . Insect Biochem Mol Biol 38: 113–123. 18070670 10.1016/j.ibmb.2007.09.007

[31] Feyereisen R (2005) Insect cytochrome P450. In: Gilbert LI, Iatrou K, Gill SS, editors. Comprehensive Molecular Insect Science. Boston: Elsevier. pp. 1–77.

[32] Li X, Schuler MA, Berenbaum MR (2007) Molecular mechanisms of metabolic resistance to synthetic and natural xenobiotics. Ann Rev Entomol 52: 231–253. 16925478 10.1146/annurev.ento.51.110104.151104

[33] Scott JG, Kasai S (2001) Monooxygenase-mediated insecticide resistance: regulation of CYP6D1 expression. In: Clark J, Yamaguchi I, editors. Agrochemical Resistance: Extent, Mechanism, and Detection. Washington, DC: American Chemical Society (ACS Symposium Series). pp. 24–41.

[34] Tomita T, Scott JG (1995) cDNA and deduced protein sequence of *CYP6D1*: the putative gene for a cytochrome P450 responsible for pyrethroid resistance in house fly. Insect Biochem Mol Biol 25: 275–283. 7711755 10.1016/0965-1748(94)00066-q

[35] Amichot M, Tarés S, Brun-Barale A, Arthaud L, Bride JM, et al (2004) Point mutations associated with insecticide resistance in the *Drosophila* cytochrome P450 *Cyp6a2* enable DDT metabolism. Eur J Biochem 271: 1250–1257. 15030474 10.1111/j.1432-1033.2004.04025.x

[36] Daborn PJ, Yen JL, Bogwitz MR, Le Goff G, Feil E, et al (2002) A single P450 allele associated with insecticide resistance in *Drosophila* . Science 297: 2253–2256. 12351787 10.1126/science.1074170

[37] Komagata O, Kasai S, Tomita T (2010) Overexpression of cytochrome P450 genes in pyrethroid-resistant *Culex quinquefasciatus* . Insect Biochem Mol Biol 40: 146–152. 20080182 10.1016/j.ibmb.2010.01.006

[38] Müller P, Warr E, Stevenson BJ, Pignatelli P, Morgan JC, et al (2008) Field-caught permethrin-resistant *Anopheles gambiae* overexpress CYP6P3, a P450 that metabolises pyrethroids. PLoS Genet 4: e1000286. 19043575 10.1371/journal.pgen.1000286 PMC2583951

[39] Ahmad M, Denholm I, Bromilow RH (2006) Delayed cuticular penetration and enhanced metabolism of deltamethrin in pyrethroid-resistant strains of *Helicoverpa armigera* from China and Pakistan. Pest manag Sci 62: 805–810. 16649192 10.1002/ps.1225

[40] Puinean AM, Foster SP, Oliphant L, Denholm I, Field LM, et al (2010) Amplification of a cytochrome P450 gene is associated with resistance to neonicotinoid insecticides in the aphid *Myzus persicae* . PLoS Genet 6: e1000999. 20585623 10.1371/journal.pgen.1000999 PMC2891718

[41] Valles SM, Dong K, Brenner RJ (2000) Mechanisms responsible for cypermethrin resistance in a strain of German cockroach, *Blattella germanica* . Pestic Biochem Physiol 66: 195–205.

[42] Shono T, Ohsawa K, Casida JE (1979) Metabolism of *trans*- and *cis*-permethrin, *trans*- and *cis*-cypermethrin, and decamethrin by microsomal enzymes. J Agric Food Chem 27: 316–325. 429687 10.1021/jf60222a059

[43] Finney DJ (1971) Probit analysis 3rd ed. Cambridge: Cambridge University Press.

[44] Kasai S, Weerasinghe IS, Shono T (1998) P450 monooxygenases are an important mechanism of permethrin resistance in *Culex quinquefasciatus* Say larvae. Arch Insect Biochem Physiol 37: 47–56.

[45] Hickey W, Craig Jr GB (1966) Genetic distortion of sex ratio in a mosquito, *Aedes aegypti* . Genetics 53: 1177–1196. 5958915 10.1093/genetics/53.6.1177 PMC1211089

[46] Kasai S, Ng LC, Lam-Phua SG, Tang CS, Itokawa K, et al (2011) First detection of a putative knockdown resistance gene in major mosquito vector, *Aedes albopictus* . Jpn J Infect Dis 64: 217–221. 21617306

[47] Funaki E, Dauterman WC, Motoyama N (1994) *In vitro* and *in vivo* metabolism of fenvalerate in pyrethroid resistant houseflies. J Pesticide Sci 19: 43–52.

[48] Mahmood T, Funaki E, Y K, Motoyama N (1993) *In vivo* studies on the mechanism of pyrethroid resistance in the German cockroach. J Pesticide Sci 18: 271–276.

[49] Lee SST, Scott JG (1989) An improved method for preparation, stabilization, and storage of house fly (Diptera: Musciadae) microsomes. J Econ Entomol 82: 1559–1563. 2607028 10.1093/jee/82.6.1559

[50] Livak KJ, Schmittgen TD (2001) Analysis of relative gene expression data using real-time quantitative PCR and 2^−ΔΔCt^ method. Methods 25: 402–408. 11846609 10.1006/meth.2001.1262

[51] Stone BF (1968) A formula for determining degree of dominance in case of monofactorial inheritance of resistance to chemicals. Bull World Health Organ 38: 325–326. 5302309 PMC2554319

[52] Kennaugh L, Pearce D, Daly JC, Hobbs AA (1993) A piperonyl butoxide synergizable resistance to permethrin in *Helicoverpa armigera* which is not due to increased detoxification by cytochrome P450. Pestic Biochem Physiol 45: 234–241.

[53] Sun YP, Johnson ER (1972) Quasi-synergism and penetration of insecticides. J Econ Entomol 65: 349–353. 5016658 10.1093/jee/65.2.349

[54] Gunning RV, Devonshire AL, Moores GD (1995) Metabolism of esfenvalerate by pyrethroid-susceptible and -resistant australian *Helicoverpa armigera* (Lepidoptera: Noctuidae). Pestic Biochem Physiol 51: 205–213. 10.1006/pest.1996.0031 8980026

[55] Nene V, Wortman JR, Lawson D, Haas B, Kodira C, et al (2007) Genome sequence of *Aedes aegypti*, a major arbovirus vector. Sicnece 316: 1718–1723. 10.1126/science.1138878 PMC2868357 17510324

[56] Saavedra-Rodriguez K, Suarez AF, Salas IF, Strode C, Ranson H, et al (2012) Transcription of detoxification genes after permethrin selection in the mosquito *Aedes aegypti* . Insect Mol Biol 21: 61–77. 22032702 10.1111/j.1365-2583.2011.01113.x PMC3540788

[57] Bariami V, Jones CM, Poupardin R, Vontas J, Ranson H (2012) Gene amplification, ABC transporters and cytochrome P450s: unraveling the molecular basis of pyrethroid resistance in the dengue vector, *Aedes aegypti* . PLoS Neglected Tropical Diseases 6: e1692. 22720108 10.1371/journal.pntd.0001692 PMC3373657

[58] Poupardin R, Riaz MA, Jones CM, Chandor-Proust A, Reynaud S, et al (2012) Do pollutants affect insecticide-driven gene selection in mosquitoes? Experimental evidence from transcriptomics. Aquat Toxicol 114–115: 114–115. 10.1016/j.aquatox.2012.02.001 22406618

[59] Stevenson BJ, Pignatelli P, Nikou D, Paine MJI (2012) Pinpointing P450s associated with pyrethroid metabolism in the dengue vector, *Aedes aegypti*: developing new tools to combat insecticide resistance. PLoS Neglected Tropical Diseases 63: e1595. 10.1371/journal.pntd.0001595 PMC3313934 22479665

[60] Hardstone MC, Leichter C, Harington LC, Kasai S, Tomita T, et al (2007) Cytochrome P450 monooxygenase-mediated permethrin resistance confers limited and larval specific cross-resistance in the southern house mosquito, *Culex pipiens quinquefasciatus* . Pestic Biochem Physiol 89: 175–184.

[61] Wilding CS, Smith I, Lynd A, Yawson AE, Weetman D, et al (2012) A cis-regulatory sequence driving metabolic insecticide resistance in mosquitoes: Functional characterisation and signatures of selection. Insect Biochem Mol Biol 42: 699–707. 22732326 10.1016/j.ibmb.2012.06.003

[62] Itokawa K, Komagata O, Kasai S, Okamura Y, Masada M, et al (2010) Genomic structures of *Cyp9m10* in pyrethroid resistant and susceptible strains of *Culex quinquefasciatus* . Insect Biochem Mol Biol 40: 631–640. 20600899 10.1016/j.ibmb.2010.06.001

[63] Itokawa K, Komagata O, Kasai S, Masada M, Tomita T (2011) *Cis*-acting mutation and duplication: History of mlecular evolution in a P450 haplotype responsible for insecticide resistance in *Culex quinquefasciatus* . Insect Biochem Mol Biol 41: 503–512. 21540111 10.1016/j.ibmb.2011.04.002

[64] Sanchez-Arroyo H, Koehler PG, Valles SM (2001) Effects of the synergists piperonyl butoxide and *S*,*S*,*S*-tributyl phosphorotrithioate on propoxure pharmacokinetics in *Blattella germanica* (Blattodea: Blattellidae). J Econ Entomol 94: 1209–1216. 11681686 10.1603/0022-0493-94.5.1209

[65] Scott JG, Georghiou GP (1986) Mechanisms responsible for high levels of permethrin resistance in the house fly. Pestic Sci 17: 195–206.

[66] Winteringham FPW, Harrison A, Bridges PM (1955) Absorption and metabolism of [^14^C]pyrethroids by the adult housefly, *Musca domestica* L., *in vivo* . Biochem J 61: 359–367. 13269368 10.1042/bj0610359 PMC1215798

[67] Bull DL, Pryor NW (1990) *In vivo* and *in vitro* fate of fenvalerate in house flies. Pestic Biochem Physiol 38: 140–152.

[68] Shono T, Unai T, Casida JE (1978) Metabolism of permethrin isomers in American cockroach adults, house fly adults, and cabbage looper larvae. Pestic Biochem Physiol 9: 96–106.

[69] Oakeshott JG, Claudianos C, Campbell PM, Newcomb RD, Russell RJ (2005) Biochemical genetics and genomics of insect esterases. In: Gilbert L, Iatrou K, Gill S, editors. Comprehensive Molecular Insect Science. Oxford, UK: Elsevier. pp. 309–361.

[70] Field LM, Blackman RL (2003) Insecticide resistance in the aphid *Myzus persicae* (Sulzer): chromosome location and epigenetic effects on esterase gene exression in clonal lineages. Biol J Linnean Soc 79: 107–113.

[71] Field LM (2000) Methylation and expression of amplified esterase genes in the aphid *Myzus persicae* (Sulzer). Biochem J 349: 863–868. 10903149 10.1042/bj3490863 PMC1221215

[72] Qiao CL, Raymond M (1995) The same esterase B1 haplotype is amplified in insecticide-resistant mosquitoes of the *Culex pipiens* complex from the Americas and China. Heredity 74: 339–345. 7538988 10.1038/hdy.1995.51

[73] Schmidt JM, Good RT, Appleton B, Sherrard J, Raymant GC, et al (2010) Copy number variation and transposable elements feature in recent, ongoing adaptation at the *cyp6g1* locus. PLoS Genet 6: e1000998. 20585622 10.1371/journal.pgen.1000998 PMC2891717

[74] Itokawa K, Komagata O, Kasai S, Kawada H, Mwatele C, et al (2013) Global spread and genetic variants of the two *CYP9M10* haplotype forms associated with insecticide resistance in *Culex quinquefascaitus* Say. Heredity 111: 216–226. 23632895 10.1038/hdy.2013.40 PMC3746819

[75] Timoshevskiy VA, Severson DW, deBruyn BS, Black WC, V SI, et al (2013) An integrated linkage, chromosome, and genome map for the yellow fever mosquito *Aedes aegypti* . PLoS Negl Trop Dis 7: e2052. 23459230 10.1371/journal.pntd.0002052 PMC3573077

[76] Pavlidi N, Monastirioti M, Daborn P, Livadaras I, Van Leeuwen T, et al (2012) Transgenic expression of the *Aedes aegypti CYP9J28* confers pyrethroid resistance in *Drosophila melanogaster* . Pestic Biochem Physiol 104: 132–135.

[77] Lewin B (2000) Genes VII. New York: Oxford University Press. 990 p.

[78] Sun W, Margam VM, Sun L, Buczkowski G, Bennett GW, et al (2006) Genome-wide analysis of phenobarbital-inducible genes in *Drosophila melanogaster* . Insect Mol Biol 15: 455–464. 16907832 10.1111/j.1365-2583.2006.00662.x

[79] Willoughby L, Chung H, Lumb C, Robin C, Batterham P, et al (2006) A comparison of *Drosophila melanogaster* detoxification gene induction responses for six insecticides, caffeine and phenobarbital. Insect Biochem Mol Biol 36: 934–942. 17098168 10.1016/j.ibmb.2006.09.004

[80] Donnelly MJ, Corbel V, Weetman D, Wilding CS, Williamson MS, et al (2009) Does *kdr* genotype predict insecticide-resistance phenotype in mosquitoes? Trends Parasitol 25: 213–219. 19369117 10.1016/j.pt.2009.02.007

