# Supplementary figures and images for: Mechanisms of Pyrethroid Resistance in the Dengue Mosquito Vector, Aedes aegypti: Target Site Insensitivity, Penetration, and Metabolism
Source: PLoS Negl Trop Dis. 2014 Jun 19;8(6):e2948. doi: 10.1371/journal.pntd.0002948 (PMC4063723; doi:10.1371/journal.pntd.0002948)

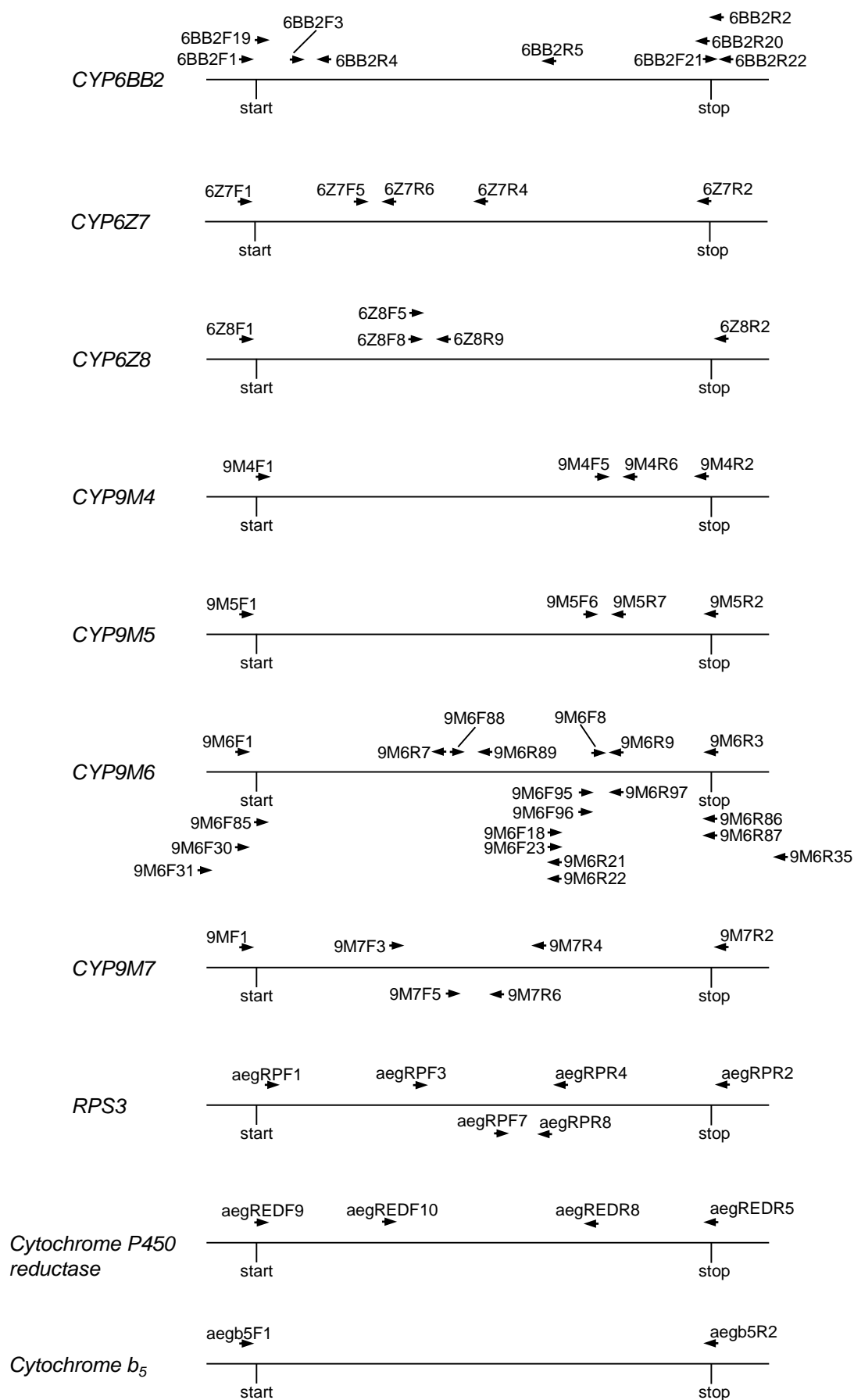

**Figure S1** Diagram depicting primer positions

Supplement: Figure S1 — Diagram depicting primer positions. (PDF) [file pntd.0002948.s001.pdf]
