# Supplementary material for: Mechanisms of Pyrethroid Resistance in the Dengue Mosquito Vector, Aedes aegypti: Target Site Insensitivity, Penetration, and Metabolism
Source: PLoS Negl Trop Dis. 2014 Jun 19;8(6):e2948. doi: 10.1371/journal.pntd.0002948 (PMC4063723; doi:10.1371/journal.pntd.0002948)
Supplement: Figure S2 — Multiple alignments of CYP9M6 cDNA sequences identified from SP and SMK strains of Aedes aegypti. Hyphens indicate identical nucleotides to the sequence of CYP9M6v1 (SP). CYP9M6v1 and v2 are identified from SP strain. CYP9M6v3, v4, and v5 were identified from SMK strain. Primers used for genotyping were also indicated. For the deduced amino acid sequences, see Figure S4. (PDF) [file pntd.0002948.s002.pdf]

9M6v1(SP)   1:ATGGTTTTGTTGGACTTGCTTGTTGTTCTGATTCCGATCGTTTCGTATCTGCTCTACCGA 60

9M6v2(SP)   1:-- 60

9M6v3(SMK)   1:---T 60

9M6v4(SMK)   1:---T 60

9M6v5(SMK)   1:---T 60

9M6v1(SP)   61:TGGGCAGTGGCCACCTACGATTTTTTTGAAAAGCGAAAAATTCCCTATGTGAAACCCTAC 120

9M6v2(SP)   61:--A--------- 120

9M6v3(SMK)   61:-- 120

9M6v4(SMK)   61:-- 120

9M6v5(SMK)   61:-- 120

9M6v1(SP)   121:CCGTTTGTTGGGGGATTGTGGCCGGTGTTCTCCGGGAAGCTCCATCCAACCGATGCCGCC 180

9M6v2(SP)   121:-- 180

9M6v3(SMK)   121:-- 180

9M6v4(SMK)   121:-- 180

9M6v5(SMK)   121:-- 180

9M6v1(SP)   181:GTTTTGGGGTACAACCTGTTCCCGGAGAATCGCTTCTCGGGATTTTTCGCATTCCGTCGG 240

9M6v2(SP)   181:--G--------- 240

9M6v3(SMK)   181:--G--------- 240

9M6v4(SMK)   181:--G--------- 240

9M6v5(SMK)   181:--G--------- 240

```
9M6v1(SP)    241:CCGGGATATCTTATCCACGATCCAGCGCTGGCGAAACAGATTATGATCAAGGATTTCGAT 300
9M6v2(SP)    241:------------------------------------------------------------ 300
9M6v3(SMK)   241:------------------------------A----------------------------- 300
9M6v4(SMK)   241:------------------------------------------------------------ 300
9M6v5(SMK)   241:------------------------------------------------------------ 300

9M6v1(SP)    301:CATTTCACCGACCATATGAATACGATTTCGGTGGATGTGGACCCATTTTCGGGCGGGCA 360
9M6v2(SP)    301:--C-----T--T-----------------------------T--------T--------- 360
9M6v3(SMK)   301:--------T--T-----------------------------T--------T--------- 360
9M6v4(SMK)   301:--C-----T--T-----------------------------T--------T--------- 360
9M6v5(SMK)   301:--------T--T-----------------------------T--------T--------- 360

9M6v1(SP)    361:CTGTTCTTCATGGATGGACAAAGATGGCGACATGGACGGTCAGGGCTAAGTCCGGCATTT 420
9M6v2(SP)    361:--------T------------C-------T-----------T------------------ 420
9M6v3(SMK)   361:--------T------------C-------T--------A--T------------------ 420
9M6v4(SMK)   361:--------T------------C-------T-----------T------------------ 420
9M6v5(SMK)   361:--------------------------------------A--T-----------A------ 420

9M6v1(SP)    421:ACGGGAAGCAAGATGAGGAACATGTTCACTTTGCTATCCAAGTATGTCGAAGGGGCCATG 480
9M6v2(SP)    421:--------------------T--------------------------------------- 480
9M6v3(SMK)   421:------------------------------------------------------------ 480
9M6v4(SMK)   421:--------------------T--------------------------------------- 480
9M6v5(SMK)   421:--------------------T--------------------------------------- 480
```

```
9M6v1(SP)   481:CAGCGGTTGGCTCAAGATGCCGGACAAAGGAAGATGGAGCTGGAGGTACGTGATCTGTTT 540
9M6v2(SP)   481:---------------------------G-----------------A-------------- 540
9M6v3(SMK)  481:---------------------------G-----------------A-------------- 540
9M6v4(SMK)  481:---------------------------G-----------------A-------------- 540
9M6v5(SMK)  481:---------------------------G-----------------A-------------- 540

                                           --------------------->F62   ----->
9M6v1(SP)   541:CAAAAGCTTGGCAATGACATCATCACGTCGATTTCGTTTGGCGTAGAAATCGATTCGGTG 600
9M6v2(SP)   541:------------------------------------------------------------ 600
9M6v3(SMK)  541:------------------------------------------------------------ 600
9M6v4(SMK)  541:------------------------------------------------------------ 600
9M6v5(SMK)  541:------------------------------------------------------------ 600

                --------------->F88
9M6v1(SP)   601:CACAATCCAAACAACGAGTTTTTCAAAAGGGGAAAGCAGTTGGCAGCCACTGGTGGTTTT 660
9M6v2(SP)   601:------------------------------------------------------------ 660
9M6v3(SMK)  601:------------------------------------------------------------ 660
9M6v4(SMK)  601:--------------------------------------------------------A--- 660
9M6v5(SMK)  601:------------------------------------------------------------ 660

                                   <--------------------R89
9M6v1(SP)   661:CAGGGACTTAAATTTTTCTTTTCGTTGGTCGTACCCGACAGCGTTTTCAAACTATTCGGG 720
9M6v2(SP)   661:------------------------------------------------------------ 720
9M6v3(SMK)  661:-----------G------------------------------------------------ 720
9M6v4(SMK)  661:------------------------------------------------------------ 720
9M6v5(SMK)  661:-----------G------------------------------------------------ 720
```

```
9M6v1(SP)   721:ATTCGATTCCTACCCAAAGAAGCGGCTGATTTTTATGTTGATGTCGTATCGAAAACTATC 780
9M6v2(SP)   721:-----G------------------------------------------------------ 780
9M6v3(SMK)  721:-----G-----------------------------------C--------A--------- 780
9M6v4(SMK)  721:-----G--------------------------------A--------------------- 780
9M6v5(SMK)  721:-----G-----------------------------------C--------A--------- 780

9M6v1(SP)   781:AAGCATCGCGAAGAGTACAAAATTGTTCGACCAGATTTTATTCATTTGTTCGTACAAGCT 840
9M6v2(SP)   781:------------------------------------------------------------ 840
9M6v3(SMK)  781:------------------------------------------------------------ 840
9M6v4(SMK)  781:------------------------------------------------------------ 840
9M6v5(SMK)  781:------------------------------------------------------------ 840

                                     -------------------->F19
9M6v1(SP)   841:CGTAAAAACGAACTAAAAGAAGAAACAGCTGATGACGAGTTGAAAAGTGCCGGATTTACC 900
9M6v2(SP)   841:--------------------G--------------------------------------- 900
9M6v3(SMK)  841:--------------------G--------------------------------------A 900
9M6v4(SMK)  841:--------------------G--------------------------------------- 900
9M6v5(SMK)  841:--------------------G--------------------------------------- 900

9M6v1(SP)   901:ACTGTTGAGGAACACATAGAAGCTTCTACAGAGAACAGCCAATATACGGATTTGGACATC 960
9M6v2(SP)   901:-----------G-----------------G------------------------------ 960
9M6v3(SMK)  901:-----------A-----------------G-----T------------------------ 960
9M6v4(SMK)  901:-----------G-----------------G------------------------------ 960
9M6v5(SMK)  901:-----------G-----------------G-----T--T--------------------- 960
```

```
9M6v1(SP)    961:ACAGCGGTCGCTGCCTCATTCTTCTTTGGAGGCATTGAAACGACTACCACGATGCTATGC 1020
9M6v2(SP)    961:--------------------------------T--------------A------------ 1020
9M6v3(SMK)   961:-----------------------------------------------A------------ 1020
9M6v4(SMK)   961:--------------------------------T--------------A------------ 1020
9M6v5(SMK)   961:-----------------------------------------------A------------ 1020

9M6v1(SP)   1021:TTCGCTCTATACGAGCTTGCAGGAAATAAAGAAGTTCAACAAAAACTTCAAGCCGAAATT 1080
9M6v2(SP)   1021:--------------A-------A---------------------G--------A------ 1080
9M6v3(SMK)  1021:------------------------------------------------------------ 1080
9M6v4(SMK)  1021:--------------A--------------------------------------T------ 1080
9M6v5(SMK)  1021:------------------------------------------------------------ 1080

9M6v1(SP)   1081:GATTCCGTCAGAAAAGAGCTGGGAGAAGGTAGTTTGACATACGAGGTGCTCCAAAAGATG 1140
9M6v2(SP)   1081:-----A--G--G--------C-------A----------------A-------G--A--- 1140
9M6v3(SMK)  1081:--------G-----------C--------------------------------G------ 1140
9M6v4(SMK)  1081:--------G-----------C--------C-----------------------G------ 1140
9M6v5(SMK)  1081:--------G--A--------C--------------------------------G------ 1140
                                                               ------------->
                                                               ------------->
9M6v1(SP)   1141:AAGTACCTCGACATGGTCGTAACGGAAACCTTACGGCGATGGCCACCCCTGGGGATAACC 1200
9M6v2(SP)   1141:--A-----A--T------------------------------------------------ 1200
9M6v3(SMK)  1141:-----------------A-----------------------------------A------ 1200
9M6v4(SMK)  1141:-----T-----T-----------------------------------------A------ 1200
9M6v5(SMK)  1141:-----------------A-----------------------------------A------ 1200
```

```
                  --->F95
                  --->F96
9M6v1(SP)   1201:AATCGAGTATGCGTCAAACCGTACACCTTCGAGGACCATGAAGGAACTAAAGTAACCATC 1260
9M6v2(SP)   1201:------------------------------------------------------------ 1260
9M6v3(SMK)  1201:--C--------------------------------------------------------- 1260
9M6v4(SMK)  1201:--C--------------------------------------------------------- 1260
9M6v5(SMK)  1201:--C--------------------------------------------------------- 1260

                        <-------------------R97
9M6v1(SP)   1261:GAAAAGGGTCAACTCATCCAGATTCCAGTTCAGTCGTTCCATCGCGATCCCAACTTCTTC 1320
9M6v2(SP)   1261:--------------------------C--------------------------------- 1320
9M6v3(SMK)  1261:--------------------------C--------------------------------- 1320
9M6v4(SMK)  1261:--------------------------C--------------------------------- 1320
9M6v5(SMK)  1261:--------------------------C--------------------------------- 1320

                                                                        <---
9M6v1(SP)   1321:CCCGATCCGTACCGTTTCGATCCGGAGCGGTTCTCCGAAGAGAACAAACACAAAATCAAT 1380
9M6v2(SP)   1321:------------------------------------------------------------ 1380
9M6v3(SMK)  1321:------------------------------------------------------------ 1380
9M6v4(SMK)  1321:--------------A--------------------------------------------- 1380
9M6v5(SMK)  1321:------------------------------------------------------------ 1380

                 <----------------R64
9M6v1(SP)   1381:CAGGATGCCTTCTTGCCGTTTGGAAGTGGACCTCGGAACTGTATCGGCTCGAGGCTGGCG 1440
9M6v2(SP)   1381:------------------------------------------------------------ 1440
9M6v3(SMK)  1381:------------------------------------------------------------ 1440
9M6v4(SMK)  1381:--------------------C---------------------------------T----- 1440
9M6v5(SMK)  1381:-----------------------------------------A------------------ 1440
```

```
9M6v1(SP)    1441:CTGATGCAAGCCAAATGTTTGCTGTACTATTTGTTCAGTGCTTTTTCGCTGGAGTATTCC 1500
9M6v2(SP)    1441:------------------------------------------------------------ 1500
9M6v3(SMK)   1441:------------------------------------------------------------ 1500
9M6v4(SMK)   1441:------------------------------------------------------------ 1500
9M6v5(SMK)   1441:------------------------------------------------------------ 1500

9M6v1(SP)    1501:GACAAGATGGACGTGCCGATCAAGCTGAACAAGATGTCGCTGACGTATACGGCGAAGAAT 1560
9M6v2(SP)    1501:------------------------------------------------------------ 1560
9M6v3(SMK)   1501:--------------------------------------A--------------------- 1560
9M6v4(SMK)   1501:------------------------------------------------------------ 1560
9M6v5(SMK)   1501:--------------------------------------A--------------------- 1560

9M6v1(SP)    1561:GGATTTTGGTTCAATTTGCTGCCGAAAAAAGTGGCTGTGTAA                   1602
9M6v2(SP)    1561:-----------------AG---------G-------------                   1602
9M6v3(SMK)   1561:-----------------AG-A-------G-------------                   1602
9M6v4(SMK)   1561:-----------------G------------------------                   1602
9M6v5(SMK)   1561:-----------------AG-A-------G-------------                   1602
```

Figure S2 Multiple alignments of *CYP9M6* cDNA sequences identified from SP and SMK strains of *Aedes aegypti*. Hyphens indicate identical nucleotides to the sequence of *CYP9M6v1* (SP). *CYP9M6v1* and *v2* are identified from SP strain. *CYP9M6v3*, *v4*, and *v5* were identified from SMK strain. Primers used for genotyping were also indicated. For the deduced amino acid sequences, see Figure S4.
