# Supplementary material for: Mechanisms of Pyrethroid Resistance in the Dengue Mosquito Vector, Aedes aegypti: Target Site Insensitivity, Penetration, and Metabolism
Source: PLoS Negl Trop Dis. 2014 Jun 19;8(6):e2948. doi: 10.1371/journal.pntd.0002948 (PMC4063723; doi:10.1371/journal.pntd.0002948)
Supplement: Figure S3 — Full length cDNA and deduced amino acid sequence of CYP6BB2 from SP and SMK strains of Aedes aegypti. The red letter indicates the only nucleotide differentiate between SP (cytosine) and SMK (thymidine) in this region. The 6BB2F21 and 6BB2R22 primers were used for genotyping. The poly(A) addition signal (aataaa) is also underlined. (PDF) [file pntd.0002948.s003.pdf]

1 ATGGACCCAGTTACGGTGATTTTGACGATTTTCGTGGGCCTAACGGGGCTCGTCTACTTCTTTCTGAGGCGGGAGCAGCAGAAATGGCCA 90
M D P V T V I L T I F V G L T G L V Y F F L R R E Q Q K W P

91 CGATTAGGCGTTCCGTTCGCGAAGAATCCTCACCTACTATTCGGGAACGTTCGAGGAATATTCCAGAAAGAGCACAGCTGCGAAATCCTC 180
R L G V P F A K N P H L L F G N V R G I F Q K E H S C E I L

181 CAGCGATTGTACTGGGAGTTCAAGGGCCGAGGATTGAAGCTCGGTGGAATTATGAACTTCTTCCAGCCGGCAGTGCTGGTTATCGATCCG 270
Q R L Y W E F K G R G L K L G G I M N F F Q P A V L V I D P

271 GAGATCAGCAAGAGTATCCTGGTAAAAGATTTCAACAAGTTTCATGATCGCGGAATTTTCGTGGATCCGGCGGGGGATCCTCTGAGTGCC 360
E I S K S I L V K D F N K F H D R G I F V D P A G D P L S A

361 AATCTGTTCAGCCTGGAGGGAGCCCAGTGGAAGGCAATGCGTACGAAGATGTCTCCGACGTTCACCTCGGGGAAGATGAAGTACATGTTC 450
N L F S L E G A Q W K A M R T K M S P T F T S G K M K Y M F

451 GAGTCGGTTCTGAACGTGGCCGAGCGGTTGAAGGACTATTTGGCGGAGAATTGCCTGAAGGAGGACATCGAGCTGAAGAACATTCTGCAG 540
E S V L N V A E R L K D Y L A E N C L K E D I E L K N I L Q

541 CGGTTTACGATGGACGTGATTGGTAATGTGGCGTTCGGGGTTGAGTGTAACTCGATTAAGAACCCGAGTTCGGAGTTCCGGTTGATGGGC 630
R F T M D V I G N V A F G V E C N S I K N P S S E F R L M G

631 TTAAAGGCGAACCGGTTTGACGGGGTAAGGTTTTTGAAGTTCTTCATCGGAGGCGCCTACAAAAACTTTGCGAAGAAGATCAAGCTGAAG 720
L K A N R F D G V R F L K F F I G G A Y K N F A K K I K L K

721 GTCGTGGAGGACGACGTTCACAAGTTCTTCATGAGTTTGGTGCACAGTACGGTGCATTACAGGGAAGGGAACAACGTGAAAAGAAACGAC 810
V V E D D V H K F F M S L V H S T V H Y R E G N N V K R N D

811 TTTTTGAATTTGCTGATGGAGATCAAGAACAAGGGAAAGTTCAGCGATGAACCCAATTCGGGCGGCGAGGGAATCACGATGAACGAGATA 900
F L N L L M E I K N K G K F S D E P N S G G E G I T M N E I

901 GCCGCGCAGTGTTTCATTTTCTTCACGGCCGGTTTCGAGACTTCGTCCACCACAATCAACTTCTGTCTGTACGAGTTGGCTAACAATCCG 990
A A Q C F I F F T A G F E T S S T T I N F C L Y E L A N N P

991 GATATTCAGGATCGATTGCGGAATGAGATTGAAGATGTAGTCGCTAAGGACGGAGGAGAGTTGAAATACGATACTCTGCTAGGTATGAAC 1080
D I Q D R L R N E I E D V V A K D G G E L K Y D T L L G M N

1081 TATCTGGATCGAGTTGTCAGTGAAACCCTACGGAAGTACTCGGCGGTGGACAACCTCTTCCGCATCTCCAACTCGCCGTACACTCCGGAC 1170
Y L D R V V S E T L R K Y S A V D N L F R I S N S P Y T P D

1171 GGCTGCAACTTCACCATCCCTGCAGGAACCCTGTTCCAAATTCCCATCCACTCGATGCACCACGATCCGGAGTACTTCCCTGACCCAGGT 1260
G C N F T I P A G T L F Q I P I H S M H H D P E Y F P D P G

```
1261  CGTTTCGATCCGGATCGATTCCTACCGGAAGTGGCCAAATCCCGACACCCATACTGCTACCTGCCCTTCGGTGAAGGACCCCGCGTTTGC  1350
      R  F  D  P  D  R  F  L  P  E  V  A  K  S  R  H  P  Y  C  Y  L  P  F  G  E  G  P  R  V  C

1351  ATCGGGATGCGATTCGGGTTGATGCAGACCAAGATCGGATTGGTTACGTTGCTGCGGAACTTCCGCTTCGGGCCCCGGTCCGAGACTCCG  1440
      I  G  M  R  F  G  L  M  Q  T  K  I  G  L  V  T  L  L  R  N  F  R  F  G  P  R  S  E  T  P

1441  GACCGATTGCAGTTTGAAGCGAAGACGTTTGTGCTGACGCCGCAGACCGGGATATACCTGAAGATTGAACCGATCGGGATATGAaggagt  1530
      D  R  L  Q  F  E  A  K  T  F  V  L  T  P  Q  T  G  I  Y  L  K  I  E  P  I  G  I  *
                                                                                     6BB2F21 primer
1531  gaytgtgtagagtagcttgcagagcgttggatctaaaaaccccaaaaaataaaggtgttaggcttccagcatccatcacagataagctat  1620
            6BB2R22 primer                          poly(A) addition signal
```

Figure S3 Full length cDNA and deduced amino acid sequence of CYP6BB2 from SP and SMK strains of *Aedes aegypti*. The red letter indicates the only nucleotide differentiate between SP (cytosine) and SMK (thymidine) in this region. The 6BB2F21 and 6BB2R22 primers were used for genotyping. The poly(A) addition signal (aataaa) is also underlined.
