# Supplementary material for: Mechanisms of Pyrethroid Resistance in the Dengue Mosquito Vector, Aedes aegypti: Target Site Insensitivity, Penetration, and Metabolism
Source: PLoS Negl Trop Dis. 2014 Jun 19;8(6):e2948. doi: 10.1371/journal.pntd.0002948 (PMC4063723; doi:10.1371/journal.pntd.0002948)
Supplement: Figure S4 — Multiple alignments of CYP9M6 amino acid sequences identified from Liverpool, SP, and SMK strains of Aedes aegypti. Hyphens indicate identical amino acids to the sequence of Liverpool strain which was used for the genome project. Asterisks indicate stop codons. (PDF) [file pntd.0002948.s004.pdf]

```
9M6 (Liverpool)    1:MVLLDLLVVLIPIVSYLLYRWAVATYDFFEKRKIPYVKPYPFVGGLWPVFSGKLHPTDAA 60
9M6v1 (SP)         1:------------------------------------------------------------ 60
9M6v2 (SP)         1:------------------------------------------------------------ 60
9M6v3 (SMK)        1:------------------------------------------------------------ 60
9M6v4 (SMK)        1:------------------------------------------------------------ 60
9M6v5 (SMK)        1:------------------------------------------------------------ 60

9M6 (Liverpool)   61:VLGYNLFPENRFSGFFAFRRPGYLIHDPALAKQIMIKDFDHFTDHMNTISVDVDPIFGRA 120
9M6v1 (SP)        61:------------------------------------------------------------ 120
9M6v2 (SP)        61:------------------------------------------------------------ 120
9M6v3 (SMK)       61:------------------------------T----------------------------- 120
9M6v4 (SMK)       61:------------------------------------------------------------ 120
9M6v5 (SMK)       61:------------------------------------------------------------ 120

9M6 (Liverpool)  121:LFFMDGQRWRHGRSGLSPAFTGSKMRNMFTLLSKYVEGAMQRLAQDAGQGKMELEIRDLF 180
9M6v1 (SP)       121:-------------------------------------------------R-----V---- 180
9M6v2 (SP)       121:------------------------------------------------------------ 180
9M6v3 (SMK)      121:------------------------------------------------------------ 180
9M6v4 (SMK)      121:------------------------------------------------------------ 180
9M6v5 (SMK)      121:------------------------------------------------------------ 180

9M6 (Liverpool)  181:QKLGNDIITSISFGVEIDSVHNPNNEFFKRGKQLAATGGFQGLKFFFSLVVPDSVFKLFG 240
9M6v1 (SP)       181:------------------------------------------------------------ 240
9M6v2 (SP)       181:------------------------------------------------------------ 240
9M6v3 (SMK)      181:------------------------------------------------------------ 240
9M6v4 (SMK)      181:------------------------------------------------------------ 240
9M6v5 (SMK)      181:------------------------------------------------------------ 240

9M6 (Liverpool)  241:IRFLPKEAADFYVDVVSKTIKHREEYKIVRPDFIHLFVQARKNELKEETADDELKSAGFT 300
9M6v1 (SP)       241:------------------------------------------------------------ 300
9M6v2 (SP)       241:------------------------------------------------------------ 300
9M6v3 (SMK)      241:------------------------------------------------------------ 300
9M6v4 (SMK)      241:------------------------------------------------------------ 300
9M6v5 (SMK)      241:------------------------------------------------------------ 300

9M6 (Liverpool)  301:TVEEHIEASTENSQYTDLDITAVAASFFFGGIETTTTMLCFALYELAGNKEVQQKLQAEI 360
9M6v1 (SP)       301:------------------------------------------------------------ 360
9M6v2 (SP)       301:-----------------------------------------------E------------ 360
9M6v3 (SMK)      301:------------------------------------------------------------ 360
9M6v4 (SMK)      301:------------------------------------------------------------ 360
9M6v5 (SMK)      301:------------------------------------------------------------ 360

9M6 (Liverpool)  361:DSVRKELGGGSLTYEVLQKMKYLDMVVTETLRRWPPLGITNRVCVKPYTFEDHEGTKVTI 420
9M6v1 (SP)       361:--------E--------------------------------------------------- 420
9M6v2 (SP)       361:--------ED-----M-------------------------------------------- 420
9M6v3 (SMK)      361:--------E--------------------------------------------------- 420
9M6v4 (SMK)      361:------------------------------------------------------------ 420
9M6v5 (SMK)      361:--------E--------------------------------------------------- 420
```

```
9M6 (Liverpool) 421:EKGQLIQIPVQSFHRDPSFFPDPYRFDPERFSEENKHKINQDAFLPFGSGPRNCIGSRLA 480
9M6v1 (SP)      421:-----------------N------------------------------------------ 480
9M6v2 (SP)      421:-----------------N------------------------------------------ 480
9M6v3 (SMK)     421:-----------------N------------------------------------------ 480
9M6v4 (SMK)     421:------------------------------------------------------------ 480
9M6v5 (SMK)     421:-----------------N-----------------------------------*       473

9M6 (Liverpool) 481:LMQAKCLLYYLFSAFSLEYSDKMDVPIKLNKMSLTYTAKNGFWFNLLPKKVAV*       533
9M6v1 (SP)      481:-----------------------------------------------------*       533
9M6v2 (SP)      481:----------------------------------------------V--R---*       533
9M6v3 (SMK)     481:----------------------------------------------V--R---*       533
9M6v4 (SMK)     481:-----------------------------------------------------*       533
9M6v5 (SMK)     473:                                                             473
```

Figure S4 Multiple alignments of CYP9M6 amino acid sequences identified from Liverpool, SP, and SMK strains of *Aedes aegypti*. Hyphens indicate identical amino acids to the sequence of Liverpool strain which was used for the genome project. Asterisks indicate stop codons.
