# Supplementary material for: Mechanisms of Pyrethroid Resistance in the Dengue Mosquito Vector, Aedes aegypti: Target Site Insensitivity, Penetration, and Metabolism
Source: PLoS Negl Trop Dis. 2014 Jun 19;8(6):e2948. doi: 10.1371/journal.pntd.0002948 (PMC4063723; doi:10.1371/journal.pntd.0002948)
Supplement: Table S2 — In vitro metabolism of [14C]-permethrin by microsome of SP and SMK strains of Aedes aegypti. (PDF) [file pntd.0002948.s006.pdf]

Table S2 *In vitro* metabolism of [^{14}C]-permethrin by microsome of SP and SMK strains of *Aedes aegypti.*

	Incubation time (min)							
	SP strain					SMK strain		
Metabolites	5	30	60	120	360	5	30	60
Permethrin	85.0 (0.87)	63.5 (3.80)	54.5 (5.29)	47.2 (7.50)	37.7 (8.32)	95.1 (0.21)	91.4 (0.46)	89.0 (0.66)
4'HO-permethrin	5.9 (0.44)	9.9 (1.03)	9.7 (1.00)	8.1 (0.64)	6.7 (0.80)	1.4 (0.04)	3.5 (0.26)	4.6 (0.30)
Origin (high polar)	6.4 (0.38)	22.9 (2.44)	32.4 (3.99)	41.9 (6.76)	53.4 (7.48)	1.7 (0.06)	3.1 (0.11)	4.4 (0.26)
Other metabolites	2.7 (0.08)	3.7 (0.33)	3.4 (0.43)	2.7 (0.09)	2.1 (0.22)	1.8 (0.15)	2.0 (0.10)	2.0 (0.11)
% of Total metabolites	15.0	36.5	45.5	52.8	62.3	4.9	8.6	11.0

Results are expressed as a percentage of the recovered dose.
All values are the mean of three replicates (±SE in parentheses).
