# Supplementary material for: Mechanisms of Pyrethroid Resistance in the Dengue Mosquito Vector, Aedes aegypti: Target Site Insensitivity, Penetration, and Metabolism
Source: PLoS Negl Trop Dis. 2014 Jun 19;8(6):e2948. doi: 10.1371/journal.pntd.0002948 (PMC4063723; doi:10.1371/journal.pntd.0002948)
Supplement: Table S3 — Metabolism of [14C]-permethrin by CYP9M6v1, CYP9M6v2, and CYP6BB2 expressed in Sf9 cells. (PDF) [file pntd.0002948.s007.pdf]

Table S3 Metablism of [^{14}C]-permethrin by CYP9M6v1, CYP9M6v2, and CYP6BB2 expressed in Sf9 cells.

Metabolite	Control	CYP9M6v1	CYP9M6v2	CYP6BB2
A	0.44 ± 0.15	2.7 ± 0.59	1.6 ± 0.69	0.53 ± 0.16
B	1.2 ± 0.19	5.3 ± 0.90	3.6 ± 0.40	1.2 ± 0.12
4'HO-permethrin	0.76 ± 0.36	5.5 ± 0.40	3.0 ± 0.17	15 ± 0.39
C	0.083 ± 0.24	1.6 ± 0.35	0.95 ± 0.22	2.7 ± 0.23
D	5.1 ± 0.55	4.2 ± 0.07	3.1 ± 0.15	4.7 ± 0.23
E (polar)	1.3 ± 0.89	9.0 ± 0.20	3.2 ± 0.30	25 ± 0.57
% of total metabolits	8.9 ± 2.1	28.4 ± 0.11	15.5 ± 0.69	48.8 ± 1.1

Results are expressed as a percentage of the recovered dose.
All values are mean±SE of three replicates.
